# Lanthanum induced B-to-Z transition in self-assembled Y-shaped branched DNA structure

**DOI:** 10.1038/srep26855

**Published:** 2016-05-31

**Authors:** Ashok K. Nayak, Aseem Mishra, Bhabani S. Jena, Barada K. Mishra, Umakanta Subudhi

**Affiliations:** 1 DNA Nanotechnology & Application Laboratory, CSIR-Institute of Minerals & Materials Technology, Bhubaneswar 751 013, India

## Abstract

Controlled conversion of right-handed B-DNA to left-handed Z-DNA is one of the greatest conformational transitions in biology. Recently, the B-Z transition has been explored from nanotechnological points of view and used as the driving machinery of many nanomechanical devices. Using a combination of CD spectroscopy, fluorescence spectroscopy, and PAGE, we demonstrate that low concentration of lanthanum chloride can mediate B-to-Z transition in self-assembled Y-shaped branched DNA (bDNA) structure. The transition is sensitive to the sequence and structure of the bDNA. Thermal melting and competitive dye binding experiments suggest that La^3+^ ions are loaded to the major and minor grooves of DNA and stabilize the Z-conformation. Our studies also show that EDTA and EtBr play an active role in reversing the transition from Z-to-B DNA.

Self-assembled branched DNA (bDNA) structures are of interest in fundamental research and have been found for practical use as components in biosensors and functional devices in DNA nanotechnology^1^,^2^,^3^,^4^. The conformational changes of DNA in response to inputs such as chemical stimuli generated by environmental cues are of immense importance for finding its biochemical application^5^,^6^,^7^. One of the most dramatic conformational transitions in biology is conversion of right-handed B-DNA to left-handed Z-DNA. Recent discoveries of Z-DNA specific proteins that bind to DNA and induce the B-to-Z transition under physiological conditions have highlighted its role in biological processes^8^,^9^,^10^,^11^,^12^. Moreover, formation of Z-DNA is believed to be strongly correlated with transcriptional activity, chromatin recombination and nucleosome positioning^13^,^14^,^15^,^16^. Z-DNA has also been implicated in various human diseases such as blood cancer, autoimmune disorder and Alzheimer’s disease.

Following the discovery of Z-DNA, studies show that B-to-Z transition requires extreme ionic strength conditions (4 M NaCl) and are largely limited to poly (GC)x or poly (CG)x sequences^17^,^18^. This view has been changed gradually over the past 30 years. Now it is known that CA and AT repeats as well as some heterogenous sequences can readily form Z-DNA in the presence of salts or metal complexes^19^,^20^,^21^,^22^,^23^,^24^,^25^. Recently, the B-Z transition has been explored from nanotechnological points of view and used as the driving machinery of many nanomechanical devices^26^,^27^,^28^. To date, the number of reported sequences that adopt the Z-DNA conformation is limited, and usually requires high salt concentrations of monovalent cations or at a considerable concentration of divalent cations such as Ca^2+^, Mg^2+^, Zn^2+^, Cd^2+^, and Ni^2+^ 29,30,31. These findings prompted us to hypothesize that trivalent cations such as rare earth elements (REEs) can induce B-to-Z transition. The large positive charge of trivalent Ln(III) ions is expected to result in stronger nucleic acid binding than observed for mono- or divalent ions. Secondly, though short stretch of sequences have been utilized as model molecules to study the B-Z transition, very few attempts have been made to observe the phenomenon in long DNA sequences, particularly in self-assembled bDNA structures. Metal and nucleic acid interaction has been a fascinating field of study with potential for advancement of structural biology and practical biotechnological applications. Particularly, REEs and their complexes have emerged as important compounds in the search for novel DNA binders and probes^32^,^33^. The application of rare earth complexes as *in vivo* biomarkers and specific binding probes has received worldwide attention. In the present work, we report for the first time that low concentration of La^3+^ can induce B-to-Z transition (Fig. 1) in self-assembled Y-shaped bDNA with sequence and structure selectivity.

In order to obtain the Y-shaped bDNA structures, various oligonucleotides were designed from the genomic sequences of *Rattus norvegicus* and were self-assembled using a method reported in our earlier work^34^. For each Y-shaped bDNA structure, three numbers of oligonucleotides were derived from the primers of different genes (β-actin, Catalase, SOD1 and SOD2) which we had earlier used for gene expression studies (Supplementary information)^35^,^36^. Since consecutive oligos have nearly 50% complementarity, self-assembly between any two oligos resulted into one half as dsDNA and other 50% have free overhangs for the self-assembly with the third oligo to yield the complete double stranded Y-shaped structure (Fig. 2). The self-assembled products have been shown by native polyacrylamide gel electrophoresis (nPAGE) whereas the B-to-Z transition, binding of various dyes and melting curve analysis have been examined by circular dichroism (CD) and fluorescence spectroscopy.

## Results and Discussion

**Figure 3:**
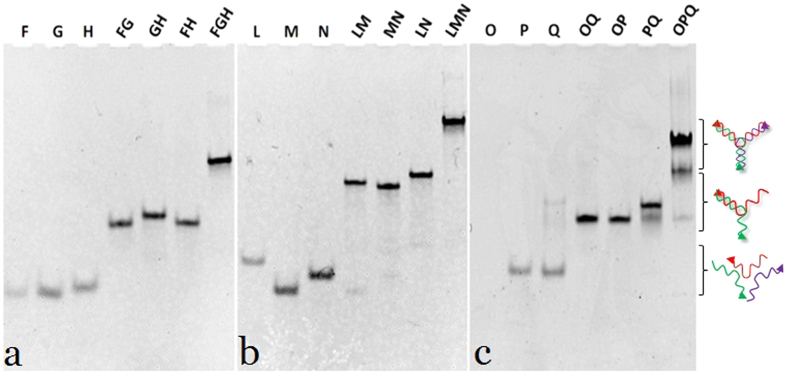
Characterization of the self-assembled bDNA structures in nPAGE (10%). The sample composition is labeled on the top of each lane. (**a**) Y-shaped bDNA structure (US1) was prepared using 40 nucleotide long oligos, (**b**) Y-shaped bDNA structure (US2) was prepared using 57 nucleotide long oligos, and (**c**) double Y-shaped structure (US3) was prepared by taking two numbers of 70 nucleotides long oligos and one short 37 nucleotides oligo. Gels showing the differential migration of individual oligos (F, G, H, L, M, N, O, P, and Q), di-oligo complexes (FG, GH, FH, LM, MN, LN, OQ, OP, and PQ), and tri-oligo complexes (FGH, LMN, and OPQ).

To confirm the self-assembly, equimolar concentration of oligos (F, G, H, L, M, N, O, P, and Q) were allowed to hybridize among each other in different combination and the differential migration of individual oligo, di-oligo complex and tri-oligo complex (Y-shaped bDNA) was observed in nPAGE (Fig. 3). Since the electrophoretic mobility of a nucleic acid oligomer or its assembly in native condition is a function of its size, shape, and extent of base pairing, the retarded mobility of DNA complexes on 10% nPAGE was used as an index of self-assembly. On the contrary, differential mobility among the di-oligo complexes is because of differential structural conformations of the assembled bDNAs. However, the yield as well as the purity of Y- shaped tri-oligo complex of our preparation can be clearly seen from nPAGE. These Y-shaped bDNA structures (US1, US2 and US3) as well as monomeric (US4) and polymeric (US5) bDNA structures of our earlier work^34^ were used for the B-Z transition study (Fig. S1).

**Figure 4:**
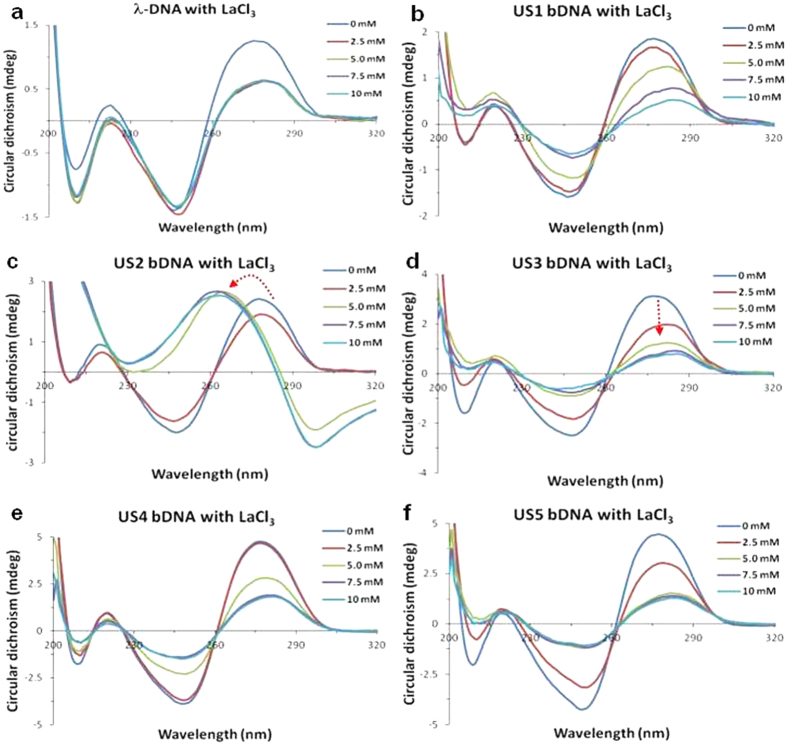
CD spectra of λ-DNA and different bDNA structures with and without LaCl_3_. All DNA samples showed typical B-DNA conformation and after addition of LaCl_3_ condensation of DNA was noticed in all (**a,b,d–f**) except US2 bDNA. A clear B-Z transition was observed only with US2 at ≥5 mM concentration (**c**).

The B-Z transition in DNA is accompanied with distinct optical signatures in the circular dichrosim (CD) spectrum emerging from the chiral nature of the molecule and the inherently different helical twist of double helix of DNA in the two conformations^37^,^38^,^39^. To start with, we conducted few experiments to understand the interaction pattern of chloride salt of REEs (LaCl_3_, GdCl_3_ and DyCl_3_) with λ-DNA. Figure S2 is a depiction of typical CD spectra of B-DNA (blue line) characterized by a positive signal at ∼280 nm and a negative signal at ∼250 nm in the absence of REEs^40^. On addition of REEs to λ-DNA a characteristic decrease in positive peak at 280 nm was observed without any change in negative peak, which is indicative of DNA condensation (Fig. S2)^41^. The degree of condensation was highest with La^3+^ and least with Dy^3+^. Possibly the binding affinity of La^3+^ is more and the binding efficiency slowly decreases with increasing atomic mass number in lanthanide series. Similar interaction was followed with different self-assembled bDNA structures (US1, US2, US3, US4, and US5). Invariably, all these bDNA structures showed characteristic B-DNA conformation with a positive peak between 275 to 280 nm and a negative peak at 250 nm (Fig. 4). The addition of LaCl_3_ resulted in decrease of both positive and negative peak in all the bDNA structures, which accounts for DNA condensation^41^. Similar effect was also observed with other REEs (Figs S3–S7). Nevertheless, the rate of condensation following the addition of REEs was different to each structure. Though condensation was recorded upto 10 mM of REEs, invariably the process slowed down after 5 mM of REEs. Commonly, binding strength between DNA and complexes parallels the magnitude of the hypochromism^42^,^43^,^44^,^45^. Interestingly, the hypochromicity and red-shift emerging in the absorbance spectra advocates for strong binding of the REEs to the bDNA structures (Figs S3–S7)^42^,^43^,^44^,^45^. While other bDNA structures showed typical condensation by interaction with La^3+^, Gd^3+^ or Dy^3+^, with increasing the concentration of LaCl_3_, US2 bDNA showed a dramatic alteration of the spectra with a characteristic positive peak at 262 nm and a negative peak at 298 nm, that is indicative of B-Z transition (Fig. 4c). Similar kind of CD signature of B-Z transition was noticed previously with different repeat sequences^46^,^47^. The Z-DNA peaks of US2 bDNA were not further influenced after gradual addition of LaCl_3_. As the B-Z transition was accompanied by a change in the UV spectrum, a marked hypochromism and bathochromic shift was also observed (Fig. S4). As shown in Fig. S8, the inversion of the CD spectrum corresponding to the B to Z transition of US2 coincides with the appearance of the bisignate CD spectrum as a function of LaCl_3_ concentration. All other bDNAs persisted in their B-form on addition of LaCl_3_ under the same conditions. It is important to note that neither Gd^3+^ nor Dy^3+^ could induce B-Z transition in US2 or any other bDNA structures under the same experimental conditions (Figs S3–S7). US2 was subsequently studied for its detail characterization including binding and melting curve analysis. As indicated previously, two factors are mainly required to trigger B-Z transition, shielding of the electrostatic repulsion from phosphate groups of DNA and reducing water activity in Z-DNA stabilization^48^,^49^,^50^. The preference for B- over Z-DNA has been ascribed to electrostatic repulsion between adjacent phosphate oxygen which are approximately 1 Å closer in the Z-form^51^. Therefore, the net result of B-Z transition was that the phosphate groups were closer together in Z-DNA than B-DNA. Under standard cellular conditions, the electrostatic repulsion of these charged phosphate groups would push the molecules into the B-DNA conformation. In the presence of high salt of monovalent cations or low concentration of LaCl_3_, the electrostatic repulsion of the phosphate residues is vastly decreased and DNA hydration layer is disturbed, hence stabilizing the Z-DNA conformation. To test whether other monovalent or divalent cations are able to mediate this transition, we chose NaCl, CaCl_2_ and BaCl_2_. From Fig. S9 it was clearly observed that, all these cations were able to condense the US2 bDNA structure, however the rate of condensation was not significant as compared to LaCl_3_. Ba is one atomic number less than La, but failed to induce B-Z transition. Even higher concentration was used to see any better condensation or transition but we failed until 100 mM. Usually higher concentration of NaCl is used for the B-Z transition, therefore 4 M NaCl was applied to the US2 bDNA, which resulted only a rapid condensation without B-to-Z transition. The ability of a cation to bind specifically to nucleic acids depends on its charge density, hydration free energy and its coordination geometry. In this regard La being an inner transition metal with higher charge density (+3) and high coordination number of eight, it can bind 8 to 9 water molecules in aqueous environment, thereby resulting high hydration free energy. This feature of La accounted for the B-to-Z transition in self-assembled bDNA structure than any other monovalent or divalent cations.

We were then interested to find whether the sequences of US2 are unique to demonstrate this transition or minor sequence modification can alter the behavior. In the control preparation wherein individual strands (L, M, and N), or di-oligo complexes (LM, MN, and LN) were interacted with LaCl_3_, only condensation was noticed without B-Z transition (Fig. S10). Now, the primary sequences of US2 are partially modified either in the end and/or middle part to obtain nine different oligos (Supplementary Information). These oligos were self-assembled to form bDNA structures and revealed by nPAGE (Fig. S11). When the four nucleotide overhangs were removed, B-Z transition disappeared (Fig. S12). Instead the positive peak at 280 nm increased to four fold which showed some abnormal conformation of the bDNA. When internal loop length was reduced to one T and overhangs were omitted only DNA condensation was revealed even after 10 mM LaCl_3_. Similarly, a more periodic condensation was observed with the intact internal sequences while around 13 nucleotides were removed from the overhang side and 9 nucleotides from other external regions. Possibly by changing the sequences and reducing the length it affects the overall conformation of B-bDNA thereby resistant to B-to-Z transition. A detailed study is necessary to decipher sequence effect and help to obtain minimal sequences that are sensitive to B-to-Z transition. From the above observations, we find that the designed bDNA US2 offers a unique example of inducible B-Z transition.

**Figure 5:**
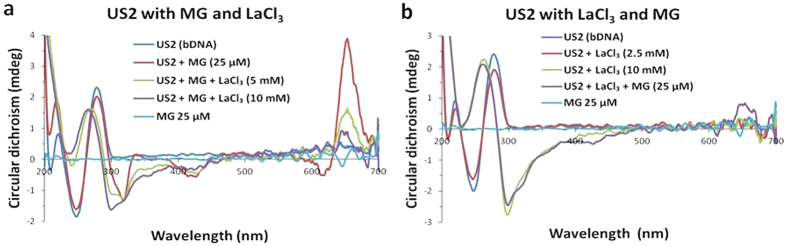
(**a**) CD spectra of bDNA US2 alone (blue), US2 with MG (red), 5 and 10 mM of LaCl_3_ to the US2-MG complex (green and violet). (**b**) CD spectra of bDNA US2 alone (blue), US2 with 2.5 and 10 mM of LaCl_3_ (red and green), MG addition to US2-La^3+^ complex (violet), MG alone shown in cyan.

To understand the manner of La^3+^ binding to bDNA, we employed a well established dye binding assay^19^. We choose methyl green (MG) that binds to the major grooves of DNA. Figure 5a showed CD spectral changes in the presence of MG. For MG there was no CD signal under our experimental conditions. When it was bound to US2 B-bDNA, four induced CD signals (one positive peak at 650 nm and three negative peaks at ∼320, 425 and 620 nm) were observed which are typical characteristic of bound MG^52^. With incremental addition of La^3+^, the induced CD gradually disappeared and at ≥5 mM concentration of La^3+^, a CD spectra of Z-form of bDNA appeared with its characteristic negative peak at 298 nm and a positive peak at 262 nm. Similar kind of MG exclusion from DNA major groove was also demonstrated by SC-dots^52^. Interestingly, our experiment supports that La^3+^ could bind to the bDNA in a manner that excludes MG from the major groove. However, weak binding of MG to La-induced Z-DNA was observed from a rudimentary peak at 650 nm, which supported that drastic decrease of major grooves in bDNA after treatment with LaCl_3_ (Fig. 5b). It is important to mention that right-handed B-DNA has both major and minor grooves, but there is only a single groove in Z-DNA which is analogous to the minor groove of B-DNA^17^,^53^. This may be the reason why MG is unable to bind to the La-induced Z-DNA and produce its induced peak.

**Figure 6:**
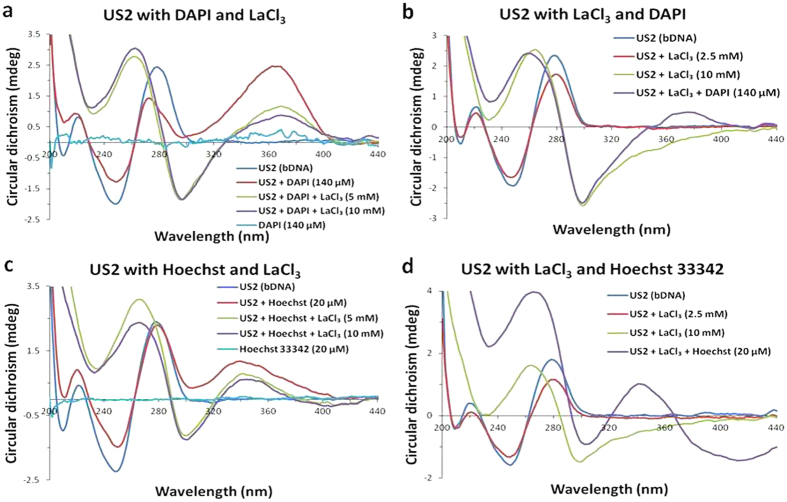
(**a,c**) CD spectra of bDNA US2 alone (blue), after interaction with DAPI or Hoechst 33342 (red), 5 and 10 mM of LaCl_3_ to the US2-DAPI or US2-Hoechst 33342 complex (green and violet, respectively), DAPI or Hoechst alone are shown in cyan. (**b,d**) CD spectra of bDNA US2 alone (blue), US2 with 2.5 and 10 mM of LaCl_3_ (red and green, respectively), DAPI or Hoechst 33342 addition to US2-La^3+^ complex (violet).

To understand how LaCl_3_ binds to the minor groove of the US2 bDNA, two well known minor groove binders such as DAPI and Hoechst 33342 are chosen for the current experiment. DAPI and Hoechst 33342 alone did not show any CD signal under our experimental condition. However, after interaction with bDNA, a positive peak was observed at 370 and 340 nm for DAPI and Hoechst 33342, respectively (Fig. 6a,c). Addition of LaCl_3_ led to decrease in induced CD. Interestingly, with ≥5 mM LaCl_3_, B-to-Z transition was observed suggesting La^3+^ possibly replaces DAPI and Hoechst 33342. Nevertheless, it was noticed that LaCl_3_ easily displaced DAPI than Hoechst 33342. In another set of experiment, these minor groove binders were interacted with La-induced Z-DNA. It was observed that the binding affinity of DAPI was not very significant to the Z-bDNA, however a minor induced peak was observed at 370 nm (Fig. 6b). Surprisingly, with Hoechst, an intense peak was observed at 340 nm and the usual DNA peak at 262 nm was enhanced (Fig. 6d). At present it is difficult to explain the induced peak. However, it can be mentioned that the Z-conformation is more suitable to interact with Hoechst than DAPI. Nevertheless, La-induced Z-conformation of US2 bDNA is stable in presence of Hoechst and DAPI. It appears that binding affinity of La^3+^ is higher than minor groove binders. To compare these observations and understand how minor groove binders are released from B-conformation of bDNA, we undertake the fluorescence competitive binding assay.

**Figure 7:**
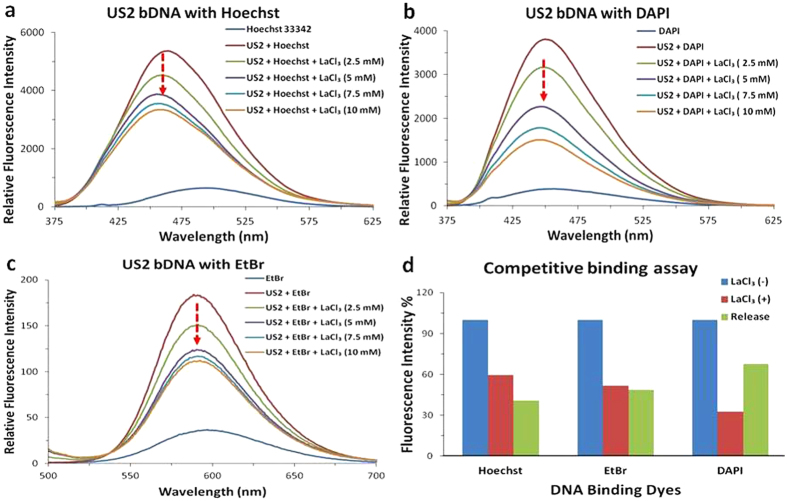
Fluorescence emission spectra of (**a**) Hoechst 33342 (excitation wavelength, 361 nm) (**b**) DAPI (excitation wavelength, 358 nm), (**c**) EtBr (excitation wavelength, 480 nm). Dye alone (blue), dye with US2 bDNA (red), different concentartion of LaCl_3_ 2.5, 5.0, 7.5 and 10 mM (green, violet, cyan, orange, respectively) added to the dye-DNA complex. (**d**) Relative fluorescence of different dye-DNA complex and its quenching by LaCl_3_.

The fluorescence quenching experiment is widely used to establish DNA binding mode^52^. In this assay DAPI, Hoechst, and ethidium bromide (EtBr) dyes were interacted with B-form of bDNA structure (US2). When DAPI and Hoechst 33342 were interacted with US2 bDNA, an enhanced fluorescence was observed (Fig. 7). Now, if La^3+^ competitively binds to the same site of bDNA where DAPI or Hoechst are bound, the fluorescence of DAPI and Hoechst would significantly decrease because the strong binding of La^3+^ with bDNA should exclude these minor groove binders. As expected when LaCl_3_ was incrementally added to this enhanced fluorescence, invariably the emission intensity decreased than the DAPI or Hoechst bound to bDNA alone (Fig. 7). With addition of 10 mM LaCl_3_, fluorescence intensity of DNA declined to 60% and 30% for Hoechst 33342 and DAPI, respectively. This is suggesting that DAPI is easily released and replaced by LaCl_3_. On the other hand, intercalating dye EtBr is widely used as a suitable fluorescence probe for DNA-binding with complexes in solution. Fluorescence quenching assay was carried out by keeping the concentration of DNA and EtBr constant while LaCl_3_ solution was added incrementally. When the solution of LaCl_3_ was added, the fluorescence intensity of bDNA-EtBr complex was gradually weakened upto 50% at about 590 nm (Fig. 7). Thus the degree of decrease in the emission intensity is parallel to the DNA binding ability of LaCl_3_ and release of EtBr molecules from DNA-EtBr hybrids into the solution. As shown in Fig. 7, when LaCl_3_ was added to US2-dye complex, the decrease in emission was highest with DAPI and lowest with Hoechst suggesting that La^3+^ has DAPI like binding. It indicated that EtBr, DAPI or Hoechst could still bind to DNA even in the presence of La^3+^. In combination with CD, competitive binding results of major and minor groove binders and fluorescence experiments further supported that La^3+^ could bind to major and minor grooves of bDNA. Nevertheless, the binding of La^3+^ is very strong towards DNA; once it is bound neither the minor groove nor major groove binders could replace and back the transition. The Z-conformation of the bDNA is retained even in the presence of minor and major groove binders.

**Figure 8:**
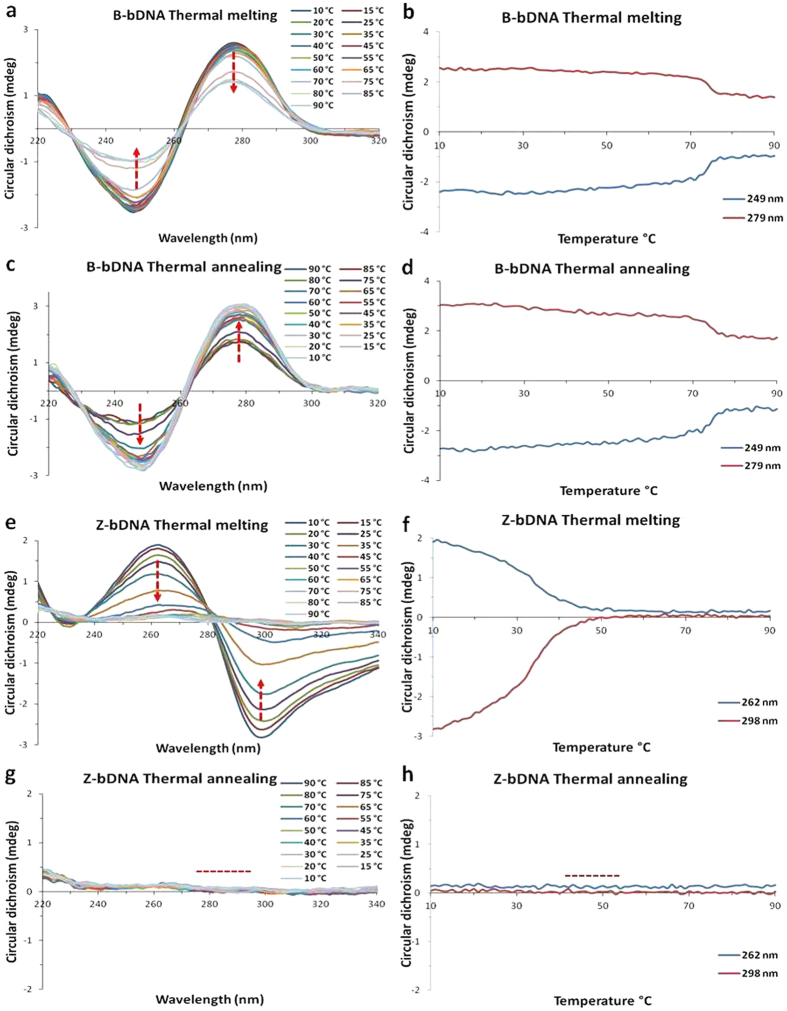
Thermal melting (**a,b**) and annealing (**c,d**) of B-form of bDNA US2 without LaCl_3_. Thermal melting (**e,f**) and annealing (**g,h**) of Z-form of bDNA US2 in presence of 10 mM LaCl_3_.

Another property of left-handed Z-DNA is its thermal instability. It has been reported that by increasing the temperature, Z-DNA slowly transforms to its right-handed B-conformation^54^. With this background, the thermal melting and annealing of US2 bDNA was carried out in the CD spectrophotometer equipped with peltier controlled accessories. The absorbance as well as CD of US2 B-bDNA was recorded as a function of temperature from 10 to 90 °C in the absence of LaCl_3_. Helix melting of US2 (B-bDNA) was monitored by following the absorbance at 260 nm, whereas the CD was recorded at 249 and 279 nm. From the thermal melting curve, it was observed that the T_m_ of US2 B-bDNA is ~75 °C in both absorbance and CD spectroscopy (Fig. 8a,b, Fig. S13). The moment temperature was reduced from 90 to 10 °C, again it went back to the normal B-conformation with the same T_m_ value (Fig. 8c,d, Fig. S13). Through CD and absorbance spectroscopy, we observed that denaturation and renaturaiton of B-conformation of US2 bDNA are reversible. On the contrary, melting curve of Z-bDNA was conducted in presence of LaCl_3_. Helix melting of US2 (Z-bDNA) was monitored by following the absorbance at 260 nm, whereas the CD was recorded at 262 and 298 nm (Fig. 8e,f, Fig. S14). From the melting curve of absorbance and CD, the T_m_ of Z-DNA was found to be ∼33 °C. The reason for the reduced T_m_ of Z-DNA can be explained as follows. The conversion of B-DNA to Z-DNA was associated with a flipping over of the base pairs so that they had an upside down orientation relative to that of B-DNA. This flipping over resulted both in the production of a syn-conformation in purine bases and a change in the deoxyribose-ring pucker in the respective bases. Moreover, in the left-handed double helix, the base pairs occupy a position at the periphery of the molecule instead of the center, as in right-handed B-DNA^20^. This may be the cause of lower T_m_ of Z-DNA, since base stacking and base pairing are not in proper order like B-DNA. Possibly bases may orient to be bound through Hoogsteen base pairing which might be an intermediate in an eventual B-to-Z transition^55^. Since the melting property depends on the hydrogen bonding among the bases, with a change in hydrogen bonding pattern directly accounts for the declined T_m_ of the Z-bDNA. With the increase in temperature both positive and negative peaks disappeared from the plot. Nevertheless, at 90 °C neither any positive peak nor negative peak of Z-bDNA was noticed. Interestingly, this distinct conformation of Z- DNA (at 90 °C) remained standstill even it was renatured upto10 °C (Fig. 8g,h, Fig. S14). Hence, the denaturation of La-induced Z-bDNA is irreversible. As mentioned earlier, to reduce the electrostatic repulsion in Z-bDNA, La^3+^ are tightly bound to the backbone of DNA. During the process of denaturation single stranded oligos are wrapped with La^3+^, which resists to renature again. Temperature alone is not able to free the bDNA from bDNA-La^3+^ complex, suggesting La^3+^ is tightly bound to the grooves as well to the phosphate backbone of DNA. Therefore, we wondered if withdrawal of La^3+^ from the La-induced Z-DNA can be achieved by some chelating agent (such as EDTA) or replaced by a strong intercalating agent like ethidium bromide it may revert back to B-conformation.

**Figure 9:**
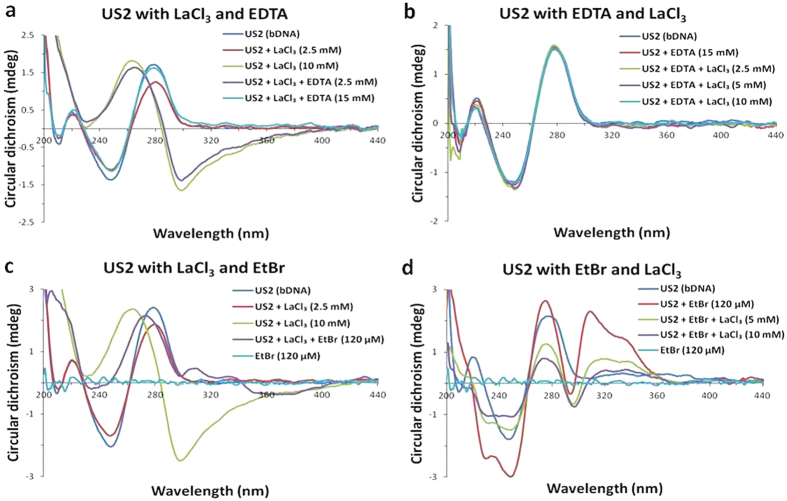
(**a**) CD spectra of bDNA US2 alone (blue), US2 with 2.5 and 10 mM of LaCl_3_ (red and green, respectively), 2.5 and 15 mM of EDTA added to US2-La^3+^ complex (violet and cyan, respectively). (**b**) CD spectra of bDNA US2 alone (blue), after interaction with EDTA (red), 2.5, 5 and 10 mM of LaCl_3_ to the US2-EDTA solution (green, violet and cyan, respectively). (**c**) CD spectra of bDNA US2 alone (blue), US2 with 2.5 and 10 mM of LaCl_3_ (red and green, respectively), 120 μM of EtBr added to US2-La^3+^ complex (violet). (**d**) CD spectra of bDNA US2 alone (blue), after interaction with EtBr (red), 5 and 10 mM of LaCl_3_ to US2-EtBr complex (green and violet, respectively). EtBr alone is shown in cyan.

EDTA, a known metal chelator was used earlier to study the reversibility of the metal ion induced B-to-Z DNA transition^56^,^57^. Once Z-DNA is achieved after interaction with LaCl_3_, the mixture was titrated with EDTA. It was noticed that with increasing concentration of EDTA the Z-conformation of bDNA slowly moves back to the B-conformation (Fig. 9a). Roughly 15 mM of EDTA was sufficient enough to reverse the process and resulted in original B-conformation of bDNA. To further verify the La^3+^ chelation mechanism by EDTA, the US2 bDNA was preincubated with EDTA and then LaCl_3_ was added. As expected in presence of EDTA no change in B-conformation was observed suggesting that before LaCl_3_ being interacting with bDNA all were trapped by EDTA, hence no B-Z transition or any other change was noticed (Fig. 9b). Generally, DNA-metal ion interactions primarily involve three binding modes: intercalative, groove and electrostatic. The intercalative binding is stronger than other two binding modes because the surface of intercalative molecule is sandwiched between the aromatic heterocyclic base pairs of DNA. Keeping this in view, La-induced Z-bDNA was treated with EtBr. After incremental addition of intercalating agent to the Z-bDNA, the characteristic negative peak at 298 nm of Z-bDNA disappeared and a significant red shift in the CD was observed with a positive peak at 275 nm (Fig. 9c). Possibly, EtBr being a strong intercalating agent was able to replace LaCl_3_ and reorganize the bases in such a way which was conducive for Z-to-B transition. A similar conversion of the left-handed form back to its right-handed conformation has been reported by EtBr^58^. However, EtBr-induced peak is missing because, there are two different types of base stacking in the left-handed helix depending on the sequence, which causes improper intercalation. While few bases are stacked on the planar bases, others interact with the O1′ atom of an adjacent sugar ring^17^,^53^. As a result, the standard base stacking of B-DNA is missing. On the contrary, EtBr binds strongly to the B-form of bDNA with a characteristic positive peak at 310 nm (Fig. 9d). Nevertheless, the prior presence of EtBr did not allow LaCl_3_ to interact with the bases and bring an upside down rotation among bases to go through the B-Z transition. However, with increased concentration of LaCl_3_ a drastic condensation was observed in the bDNA structure suggesting La^3+^ is able to bind to the minor and major grooves as well to the phosphate backbone in the presence of EtBr. This was also verified from the fluorescence study that La^3+^ binds to EtBr-bDNA complex and reduces the emission. Hence, we believe that to stabilize the Z-conformation in US2 bDNA presence of LaCl_3_ is a prerequisite.

In summary, low concentration of lanthanum can induce a left-handed helical structure in self-assembled Y-shaped bDNA with sequence and structure selectivity. One of the most important findings in our study is that Y-shaped bDNA structure has considerable conformational flexibility which transform from right-handed B-to left-handed Z-DNA. The B-Z transition is associated with an array of La^3+^ loaded on the phosphate backbone of bDNA structure. Even though the current communication reports La-induced B-Z transition in one of the self-assembled bDNA structure, La or other REEs can be explored to various bDNA structures to find B-Z transition and their possible application.

## Methods

### Self-assembly study

Self-assembly among oligos was carried out in TAE/Mg^2+^ buffer. The TAE/Mg^2+^ buffer consisted of Tris base (40 mM, pH 8.0), acetic acid (20 mM), EDTA (2 mM) and Mg(Ac)_2_ (12.5 mM). In 25 μl reaction each oligo were combined in equimolar (25 pmol each) ratio, denatured at 95 °C for 9 min and then cooled to 4 °C with ramp rate 0.3 °C/sec using a thermal cycler (S1000, Bio-Rad). All the self-assembly reactions were performed 5 times in the same experimental condition. bDNA samples were then directly used for characterization, without further fractionation or purification.

### Native Polyacrylamide gel electrophoresis

Polyacrylamide gel electrophoresis of all self assembled bDNA samples were carried out in mini vertical gel electrophoresis system (Hoefer, SE260). 10% polyacrylamide gel was prepared by adding 7.5 ml of 30% acrylamide solution (acrylamide: bis-acrylamide :: 29:1), 450 μl of 50 X TAE buffer (Tris 2 M, pH 8.0, acetic acid 1 M, EDTA 100 mM), 2.81 ml of 100 mM Mg (Ac)_2_, 300 μl of 10% APS, 15 μl of TEMED and the volume was made up to 22.45 ml with MilliQ water (18.2 MΩ.cm). This is sufficient for making two numbers of gel slab of dimension 10 × 10 cm. Electrophoresis for all samples was conducted at 150 V and 4 °C for an appropriate time according to the nature of the sample. After electrophoresis the gels were stained in EtBr solution (0.5 μg/ml) for 1 h and then viewed and documented in gel documentation system (FluorChem E, Cell Bioscience).

### Circular dichroism (CD) analysis

Circular dichroism measurements were carried out in Chirascan spectrophotometer (Applied Photophysics). Data acquisition and analysis was done using Pro-data Chirascan software. Before starting the experiment nitrogen gas was circulated through the instrument to provide oxygen free environment during experiment. All the experiments were carried out in optical cells with path length of 1 mm. CD spectra of all samples were measured at 20 °C with scan speed of 60 nm/min, bandwidth of 1 nm and time per point of 0.5 sec. The temperature of the cell was adjusted with Industrial Chiller (CW-3000) and monitored through temperature controller (Quantum Northwest, TC-425). Each smooth CD spectra is an average of three scans subtracted from assembly buffer as blank which is also average of three scans.

### DNA melting curve analysis

Melting curve experiment of both B- and Z-DNA samples was performed in CD spectrophotometer to determine the thermal stability of the DNA samples. For melting curve analysis the sample was heated from 10 to 90 °C with a rate of 0.3 °C/min and the data was recorded at 1 nm step and with tolerance of 0.02 °C. After denaturation of the DNA sample, renaturation of the same sample was carried out from 90 to 10 °C keeping same set of parameters.

### Competitive binding assay

All fluorescence studies were performed at 25 °C using a spectrofluorimeter (*Hitachi*, Model F. 2500). All the experiments were conducted in quartz cuvette with 1 cm path length. To check the EtBr emission, 7 μM of EtBr (excitation wavelength: 480 nm) alone was taken in Tris buffer and then mixed with US2 bDNA (6 μM). Now this EtBr-DNA complex was titrated with different concentration of LaCl_3_ (2.5, 5.0, 7.5, and 10 mM). Similar experiments were conducted by taking 10 μM Hoechst 33342 (excitation wavelength: 361 nm) and 7 μM DAPI (excitation wavelength: 358 nm) with US2 bDNA (24 μM) and then titrated with different concentration of LaCl_3_ (2.5, 5.0, 7.5, and 10 mM).

This assay was also conducted using CD spectroscopy. The DNA binding dyes DAPI (140 μM), Hoechst 33342 (20 μM), methyl green (25 μM) and EtBr (120 μM) used for the current experiment which did not resulted any CD spectra, however, showed characteristic induced CD spectra when bound to DNA. First these dyes individually were added to US2 bDNA and CD spectra were collected. Then dye-bDNA complex was titrated with LaCl_3_ (2.5 mM to 10 mM). On the contrary, US2 bDNA was first titrated with LaCl_3_ (2.5 mM to 10 mM) which caused B-Z transition. Now, DNA binding dyes were added to US2 Z-DNA and recorded the CD. All the experiments were conducted at 20 °C in 1 mm cuvette. Final spectra were an average of three acquired spectra which were corrected against assembly buffer as baseline.

## Additional Information

**How to cite this article**: Nayak, A. K. *et al*. Lanthanum induced B-to-Z transition in self-assembled Y-shaped branched DNA structure. *Sci. Rep*. **6**, 26855; doi: 10.1038/srep26855 (2016).

## Supplementary Material

Supplementary Information

## Figures and Tables

**Figure 1:**
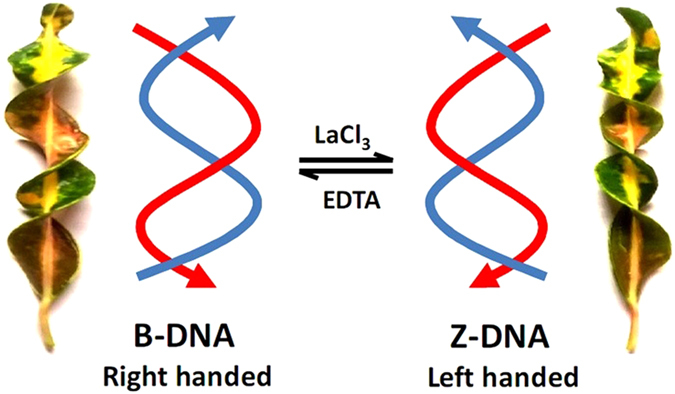
Schematic representation of B (right handed) – Z (left handed) DNA transition in presence of LaCl_3_ and reverse transition by EDTA. Natural analogy of helical structure is also presenting both right and left-handed conformation. These pictures of the Croton leaves were photographed by US (Dr Umakanta Subudhi) in his own garden during one of his early morning visits.

**Figure 2:**
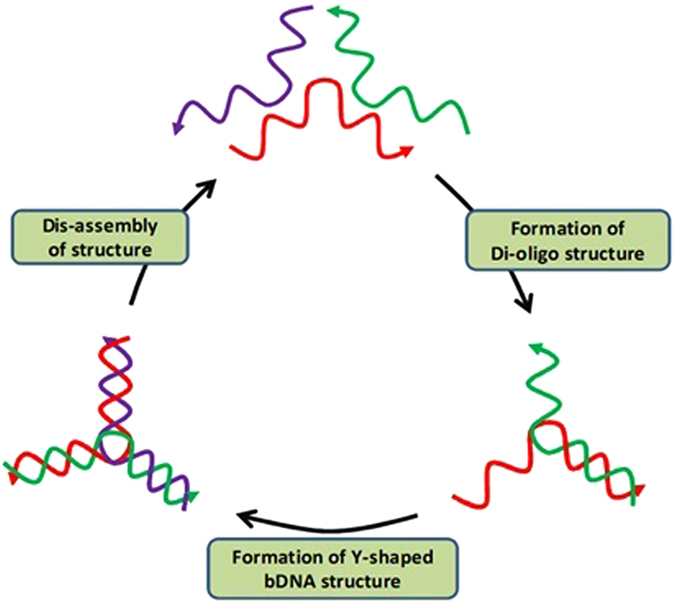
Schematic representation of self-assembly of Y-shaped bDNA structure. Individual oligos are presented in three different colors.
