# Supplementary material for: Lanthanum induced B-to-Z transition in self-assembled Y-shaped branched DNA structure
Source: Sci Rep. 2016 May 31;6:26855. doi: 10.1038/srep26855 (PMC4886512; doi:10.1038/srep26855)
Supplement: Supplementary Information [file srep26855-s1.doc]

**Supplementary Information**

**Lanthanum induced B-to-Z transition in self-assembled Y-shaped branched DNA structure**

**Ashok K Nayak^a^, Aseem Mishra^a^, Bhabani S Jena^a,b^, Barada K Mishra^a,b^, and Umakanta Subudhi^a,b,*^**

^a^CSIR-Institute of Minerals & Materials Technology, Bhubaneswar 751 013, INDIA

^b^Academy of Scientific & Innovative Research (AcSIR), New Delhi-110 025, India

***Designing of Oligonucleotides***

Oligonucleotides were designed from the forward and reverse primers of different genes such as β-actin, Cu-Zn superoxide dismutase (SOD1), Mn-superoxide dismutase (SOD2), catalase (CAT), and glyceraldehyde-3-phosphate dehydrogenase (G3PDH) of *Rattus norvegicus* (primers of these genes were used for gene expression study in our previous reports*; Chattopadhyay et al., Comp. Biochem. Physiol, 2007 and Subudhi U. and Chainy G.B.N. Mol. Biol. Rep. 2012*). For Y-shaped bDNA structures three different oligonucleotides are used. Oligonucleotides F, G and H are derived from β-actin, and SOD1 and self-assembled to form Y-shaped bDNA (US1). Any two oligos share 50% complementarity with each other. The 5′ half of oligo F has sequence complementarity to 3′ half of oligo G and 3′ half of oligo F is complementary to 5′half of oligo H. Similarly, 5′ half of oligo G assemble with 3′ half of oligo H. For Y-shaped structure (US2) three oligos L, M and N, (57 nt each) have been derived from primer sequences of CAT and SOD1. Each oligo has a common base pairing sequence (24 nt), internal loop (5T) and an 5′ overhang (4 nt). The complementary nature of oligos L, M and N is as follows. The 5′ half of oligo L binds to 3′ half of oligo M and 3′ half of oligo L binds to 5′ half of oligo N. Similarly, 5′ half of oligo M assembles with 3′ half of oligo N. Since B-Z transition was observed only in US2 bDNA, sequences of individual strands were modified for further binding to La^3+^. In this regard, 9 different oligos (R, S, T, U, V, W, X, Y and Z) are derived from oligos L, M and N. The oligos R, S and T are the result of overhang removal of oligos L, M, and N, respectively. Similarly, oligos U, V, and W have been derived after deleting both the overhang and 4T from internal loop. To generate a short stretch of sequence, 13 nt were removed from 5′ end and 9 nt were removed from the 3′ end of the primary sequence L, M, and N, which resulted oligos X, Y, and Z. However, for double Y-shaped bDNA structure (US3) three different oligos are used, those are derived from CAT and SOD1. The two longer oligos have an internal complementary region (30 nt) flanked by an external region (15 nt) and separated by thymine loop (3T). The longer oligos share base complementarity at internal region whereas the smaller oligo share complementarity at both ends of larger oligos. For the double Y-shaped structure US3, oligos O (37 nt), P, and Q (70 nt each) are used. Oligos P and Q, bear common complementary region, whereas the 5′external region of oligos P and Q share complementarity with 3′ half of oligo O. While, 3′external region of oligo P and Q binds to 5′ half of oligo O. In addition to this, monomeric unit (US4, assembled product of oligo I, B, C and D) and polymeric bDNA structure (US5, assembled product of A, B, C and D) of our earlier work (Nayak and Subudhi, RSC Advances, 2014) have been used in the B-Z transition study. All oligonucleotides were purchased in desalted form from Integrated DNA Technologies, Inc. and used without further purification.

**Name of the different bDNA structures used in the current study**

US1 Y-shaped bDNA structure (FGH)

US2 Y-shaped bDNA structure (LMN)

US3 Double Y-shaped structure (OPQ)

US4 Monomeric bDNA structure (IBCD)

US5 Polymeric bDNA structure (ABCD)

**Nucleic acid sequences of different strands**

**A**: 5’ CTG ACC GAG CGT GGC TTT CTG AGG AGA GCA GCG CTT GGC CAG CGC CTC TTT CCT GCT TGC TGA TCG 3’

**B**: 5’ CTG AGT GAC GTT GTG TTT GAG GCG CTG GCC AAG CGC TGC TCT CCT CAG TTT CGA ATG GAG AGG CAG 3’

**C**: 5’ CTG CCT CTC CAT TCG TTT TGA GCA GAA GGC AAG TAG CAG GAC AGC AGA TTT CAC AAC GTC ACT CAG 3’

**D**: 5’ GCC ACG CTC GGT CAG TTT TCT GCT GTC CTG CTA CTT GCC TTC TGC TCA TTT CGA TCA GCA AGC AGG 3’

**I**: 5’ CTC AAG ATT GTC AGC TTT CTG AGG AGA GCA GCG CTT GGC CAG CGC CTC TTT AGA TCC ACA ACG GAT 3’

**F**: 5’ TGAC AAG ATC CTG ACC GAG CGT GTT CAC CGC TTG CCT TCT 3’

**G**: 5’ TGAC CCT GCT TGC TGA TCC ACA ACG CTC GGT CAG GAT CTT 3’

**H**: 5’ TGAC AGA AGG CAA GCG GTG AAC TGT GGA TCA GCA AGC AGG 3’

**L**: 5’ GATC CTG AGT GAC GTT GTC TTC ATT AGC TTTTT TGC AGT ACA CTG CCT CTC CAT TCG 3’

**M**: 5’ GATC TAG CAG GAC AGC AGA TGA GTC TGA TTTTT GCT AAT GAA GAC AAC GTC ACT CAG 3’

**N**: 5’ GATC CGA ATG GAG AGG CAG TGT ACT GCA TTTTT TCA GAC TCA TCT GCT GTC CTG CTA 3’

**O**: 5’ AATT CAC AAC GTC ACT CAG TTT CTG CCT CTC CAT TCG 3’

**P**: 5’ GATC CGA ATG GAG AGG CAG TTT GAG GCG CTG GCC AAG CGC TGC TCT CCT CAG TTT CTG AGT GAC GTT GTG 3’

**Q**: 5’ GATC CGA ATG GAG AGG CAG TTT CTG AGG AGA GCA GCG CTT GGC CAG CGC CTC TTT CTG AGT GAC GTT GTG 3’

**Modif Modified oligos sequences of L, M, and N of US2**

**R**: 5’ CTG AGT GAC GTT GTC TTC ATT AGC TTTTT TGC AGT ACA CTG CCT CTC CAT TCG 3’

**S**: 5’ TAG CAG GAC AGC AGA TGA GTC TGA TTTTT GCT AAT GAA GAC AAC GTC ACT CAG 3’

**T**: 5’ CGA ATG GAG AGG CAG TGT ACT GCA TTTTT TCA GAC TCA TCT GCT GTC CTG CTA 3’

**U**: 5’ CTG AGT GAC GTT GTC TTC ATT AGC T TGC AGT ACA CTG CCT CTC CAT TCG 3’

**V**: 5’ TAG CAG GAC AGC AGA TGA GTC TGA T GCT AAT GAA GAC AAC GTC ACT CAG 3’

**W**: 5’ CGA ATG GAG AGG CAG TGT ACT GCA T TCA GAC TCA TCT GCT GTC CTG CTA 3’

**X**: 5’ GTT GTC TTC ATT AGC TTTTT TGC AGT ACA CTG CCT 3’

**Y**: 5’ AGC AGA TGA GTC TGA TTTTT GCT AAT GAA GAC AAC 3’

**Z**: 5’ AGG CAG TGT ACT GCA TTTTT TCA GAC TCA TCT GCT 3’

**a
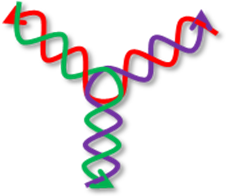
** **b
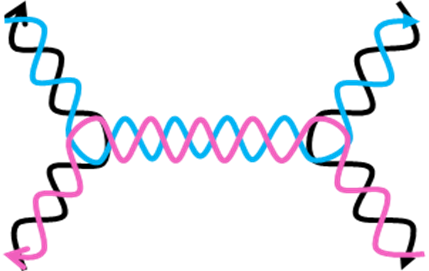
**

**c
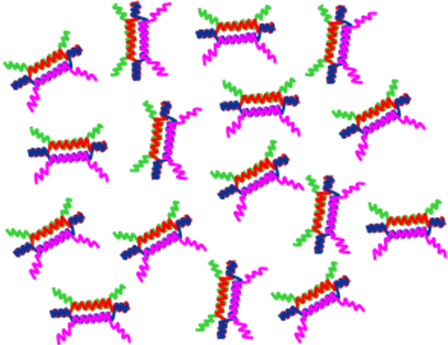
 d
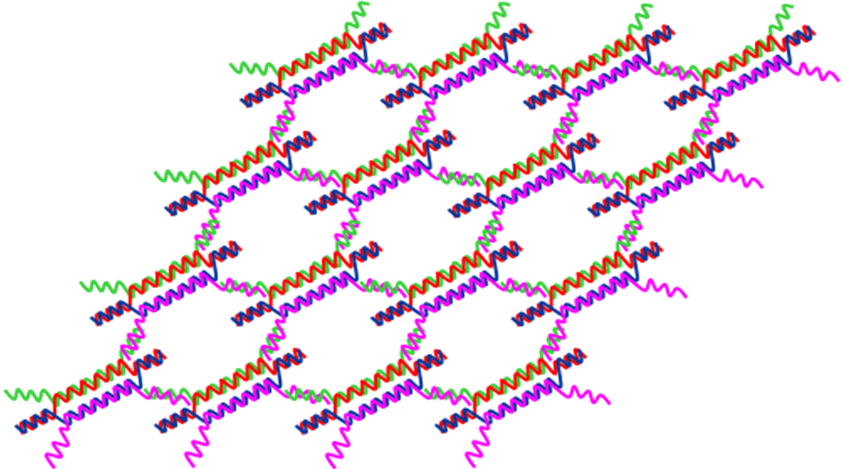
**

**Figure S1.** Schematic presentation of different bDNA structures used for the B-Z transition. (a) Simple Y-shaped structures i.e. US1 and US2, (b) double Y-shaped structure i.e. US3 (c) complex monomeric structure, US4 and (d) multiplexed polymeric bDNA structure, US5.


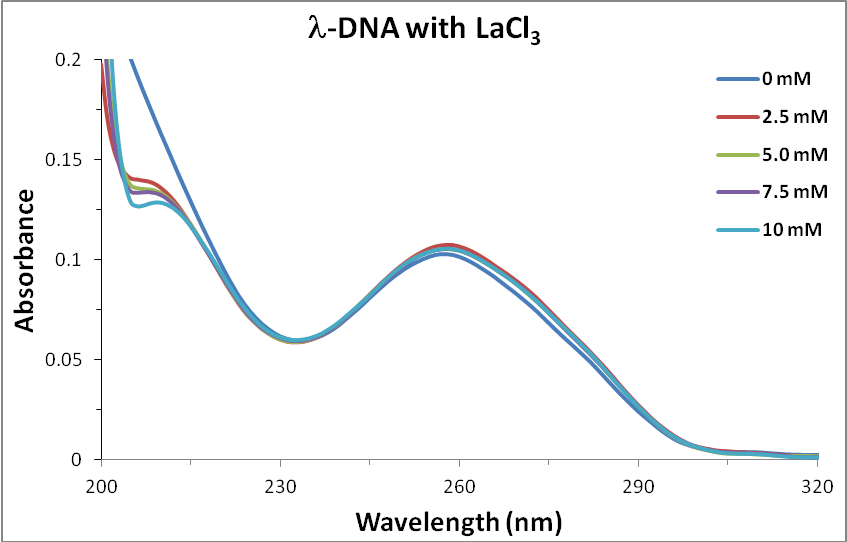

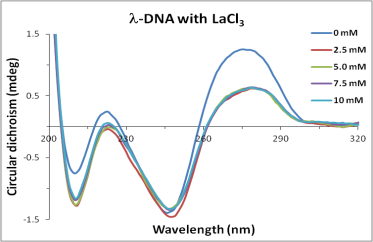

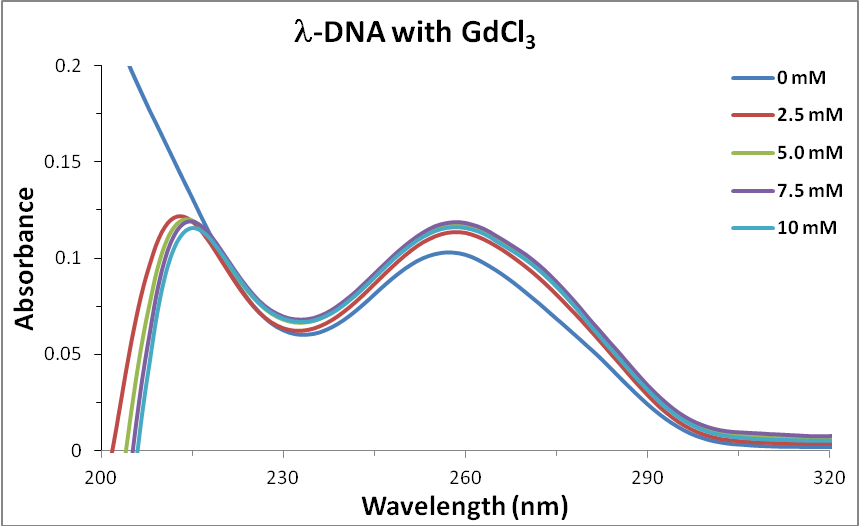

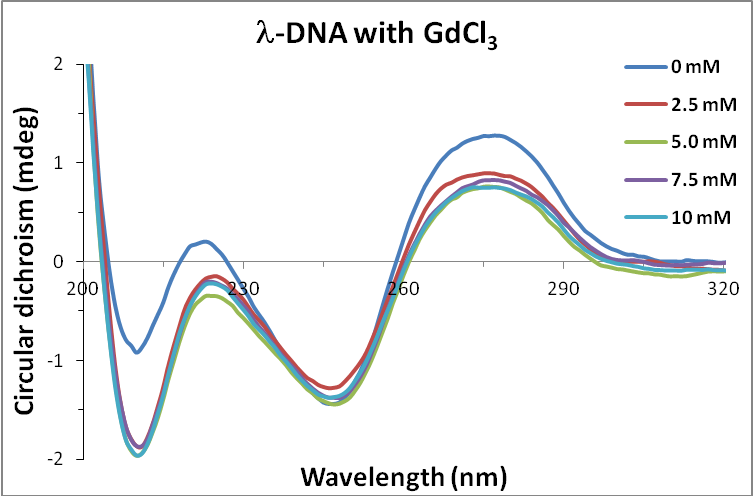

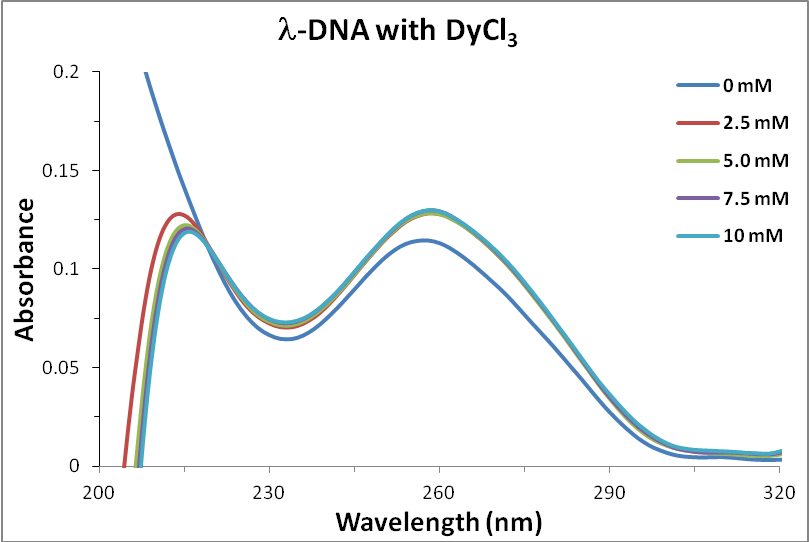

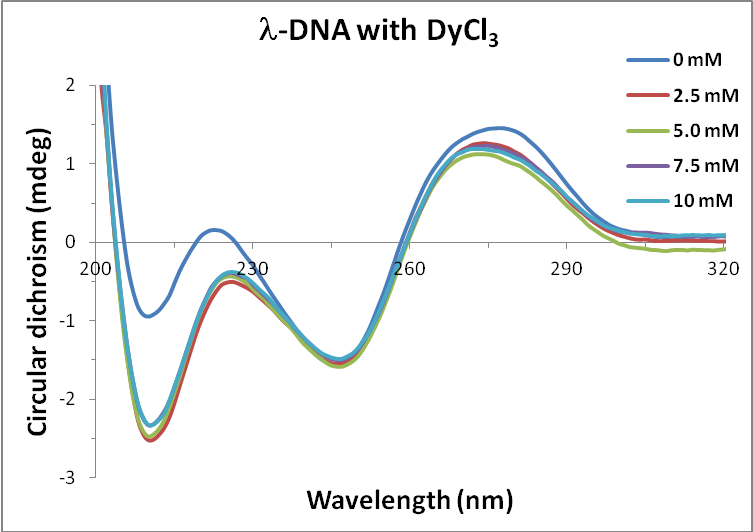


**Figure S2.** Absorbance and circular dichroism (CD) spectra of λ-DNA and its interaction with different concentrations of LaCl_3_, GdCl_3_, DyCl_3_. λ-DNA exhibit typical B-DNA conformation and DNA condensation was observed after increasing the concentration of REEs.


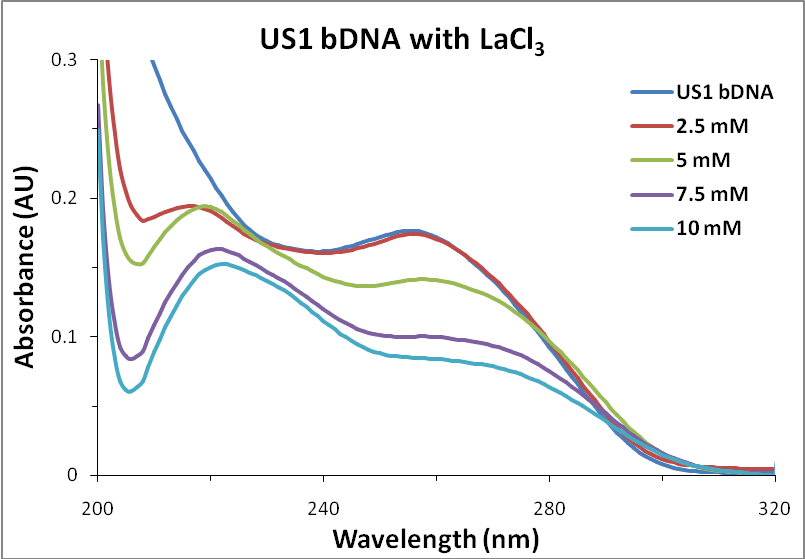

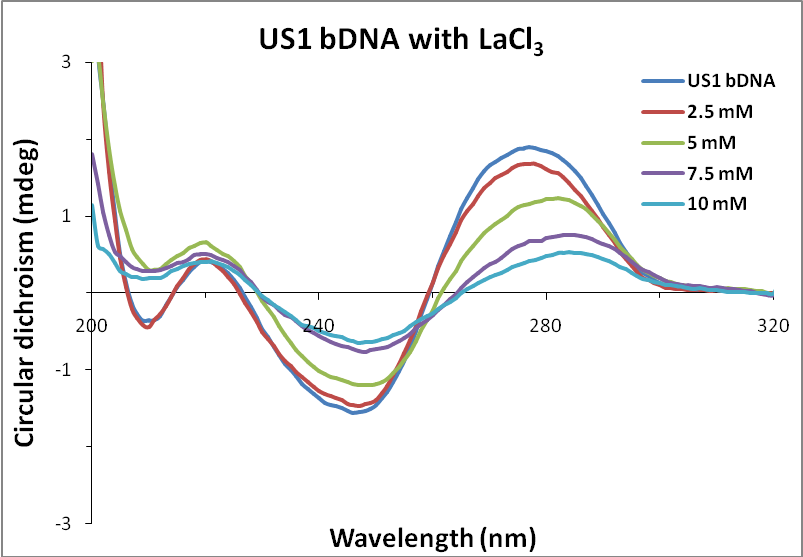

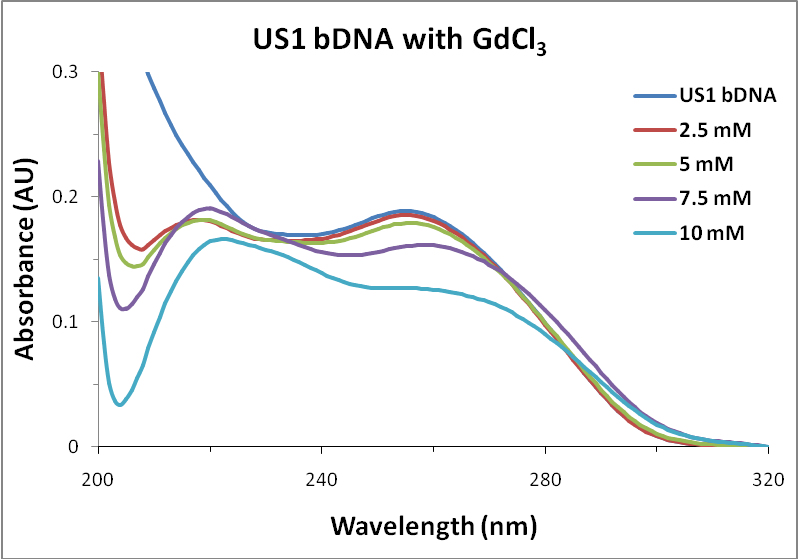

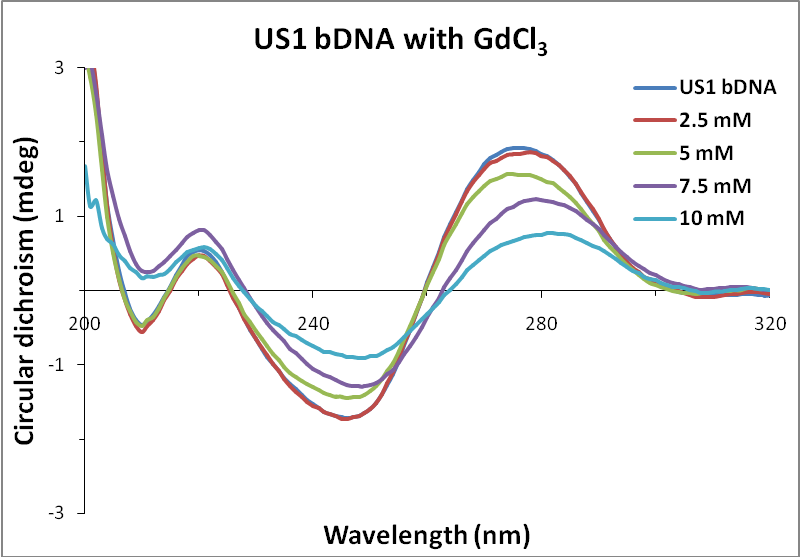

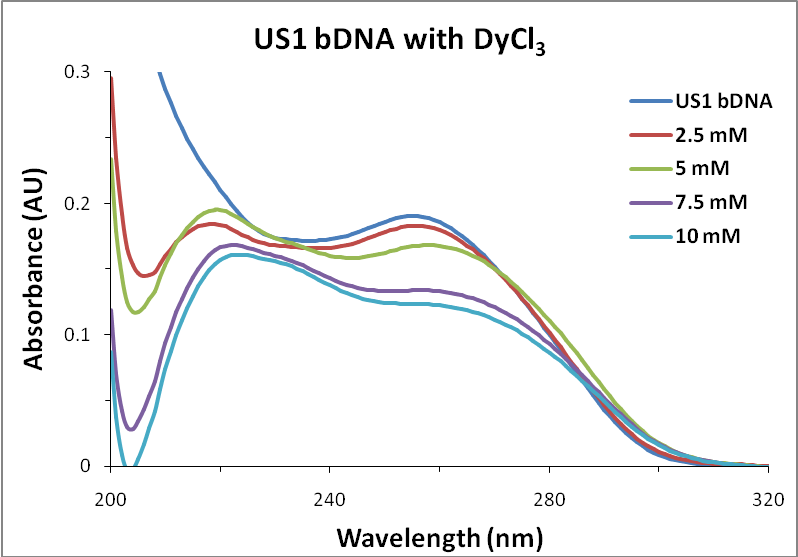

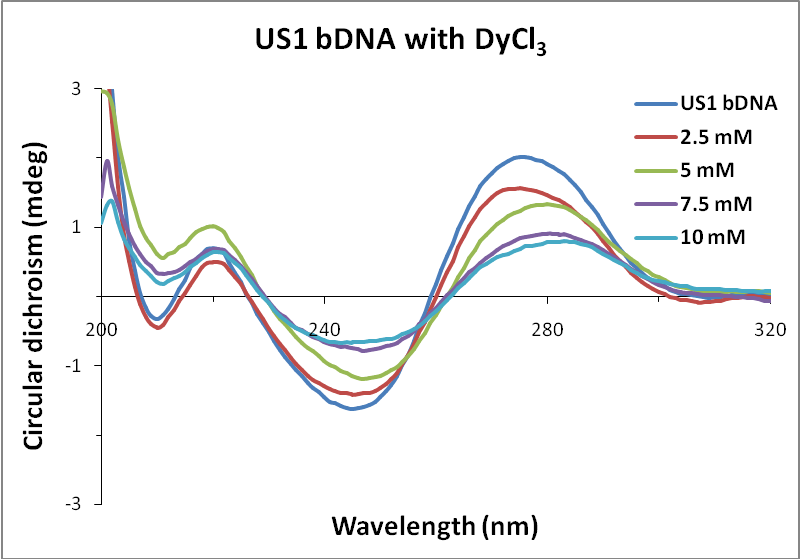


**Figure S3.** Absorbance and circular dichroism (CD) spectra of self-assembled bDNA US1 and its interaction with different concentrations of LaCl_3_, GdCl_3_, and DyCl_3_. US1 exhibit typical B-DNA conformation and DNA condensation was observed after increasing the concentration of REEs.


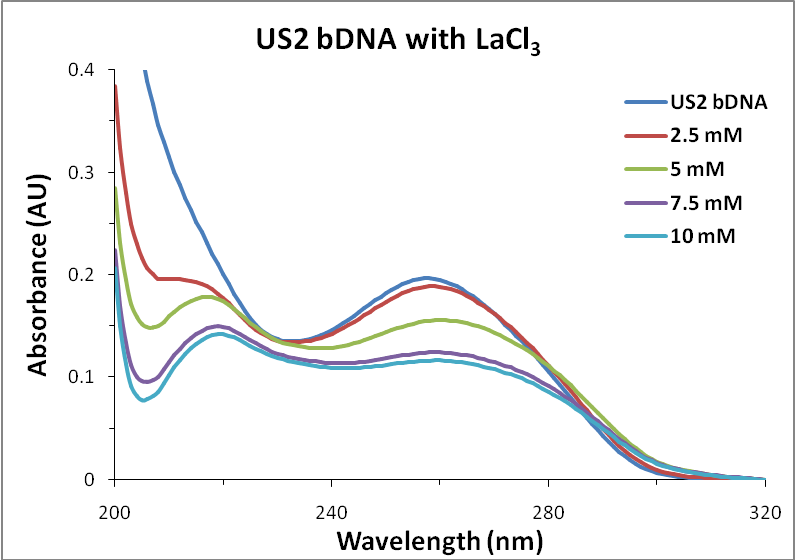

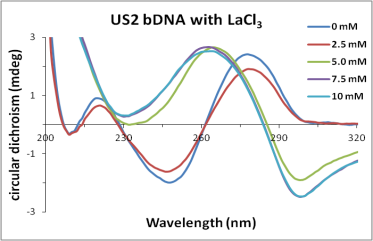

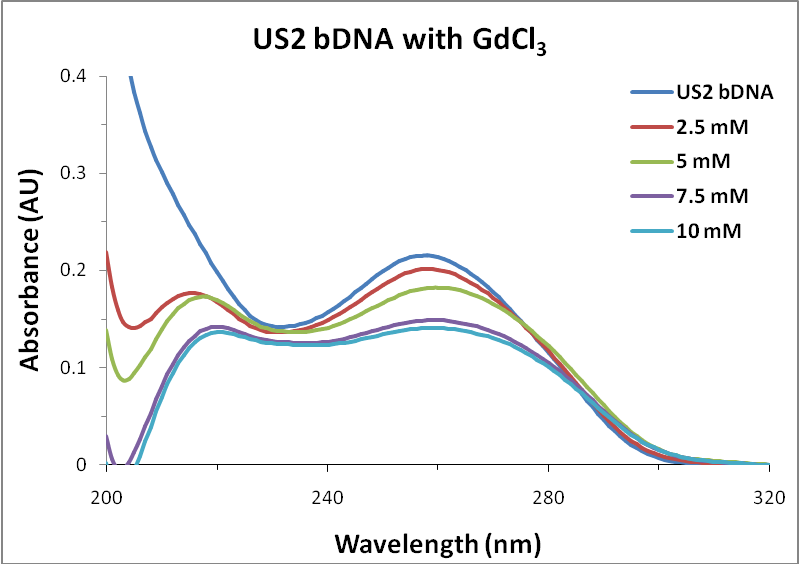

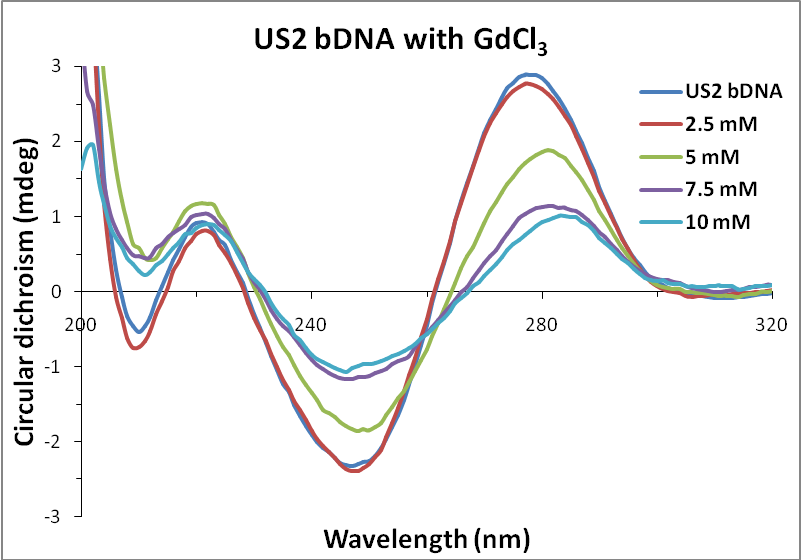

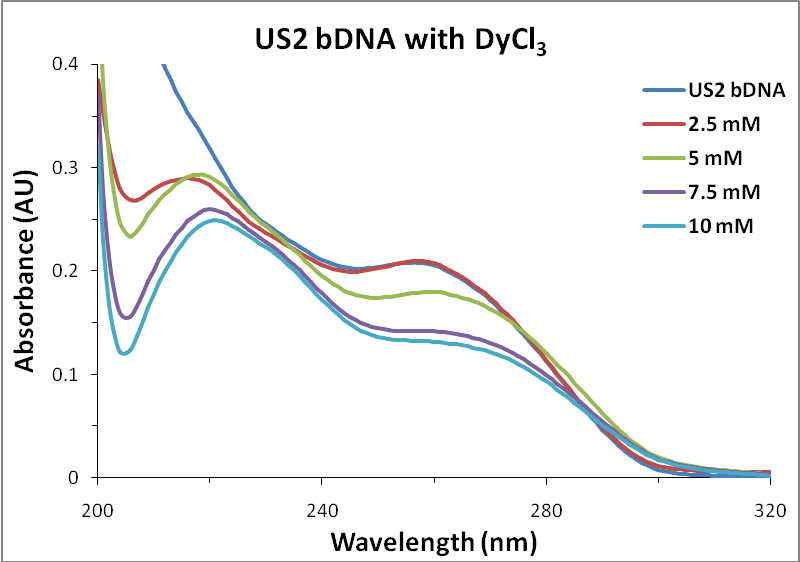

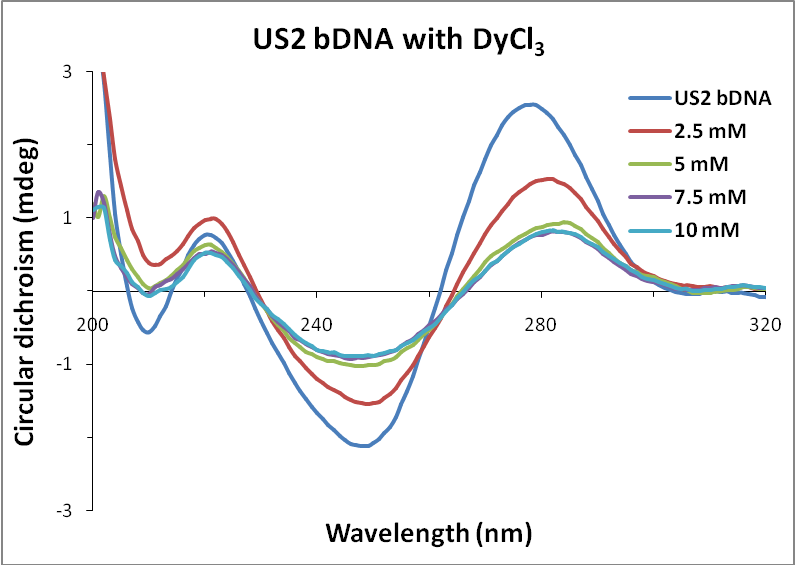


**Figure S4.** Absorbance and circular dichroism (CD) spectra of self-assembled bDNA US2 and its interaction with different concentrations of LaCl_3_, GdCl_3_, and DyCl_3_. US2 exhibit typical B-DNA conformation. The usual DNA condensation was observed after increasing the concentration of GdCl_3_, and DyCl_3_. Whereas with 5mM concentration of LaCl_3_ US2 assumed the left-handed conformation (Z-DNA) of bDNA with typical negative peak at around 298 nm and positive peak at around 263 nm.


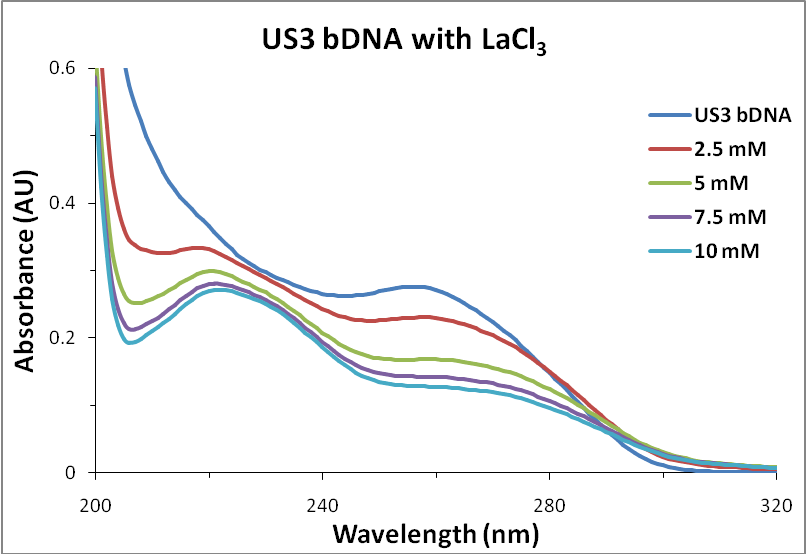

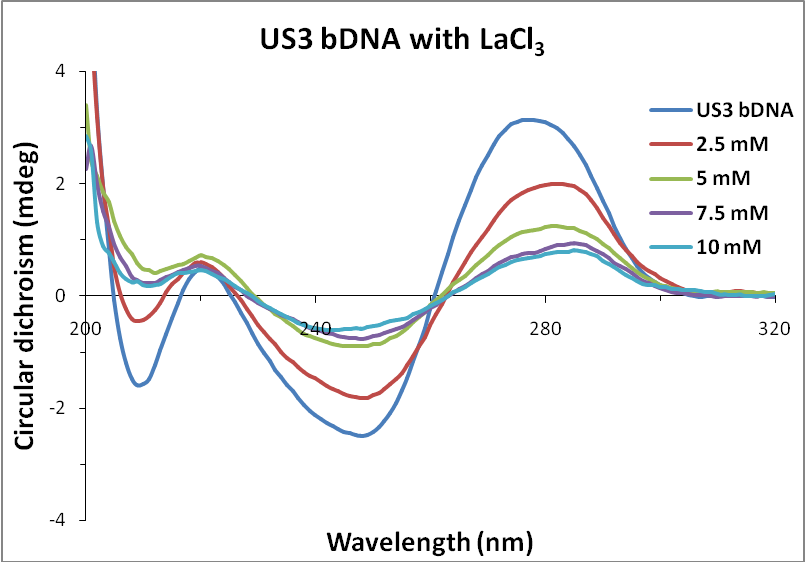

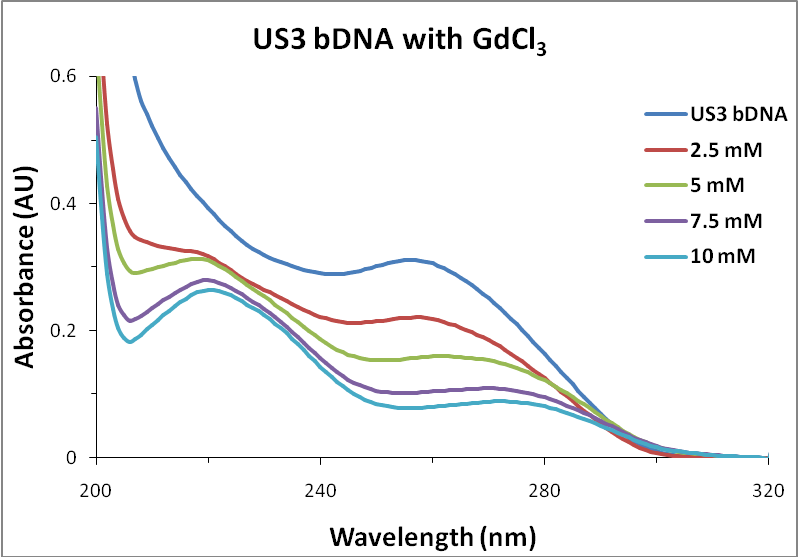

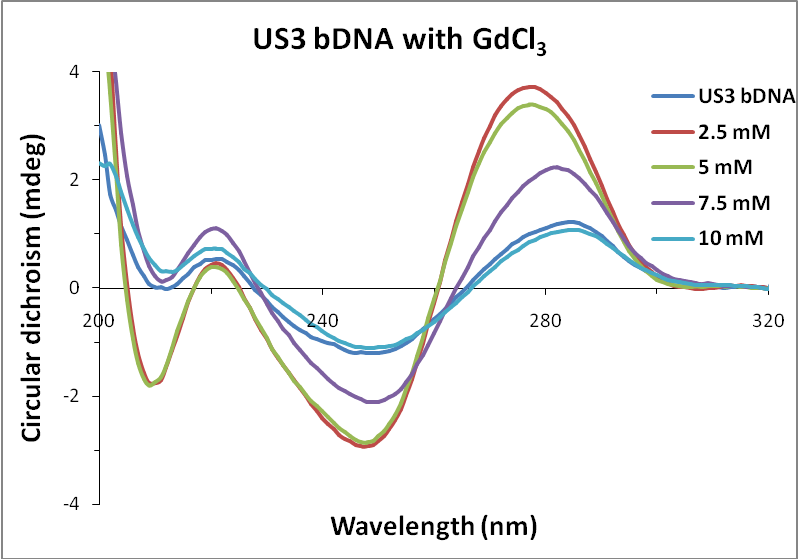

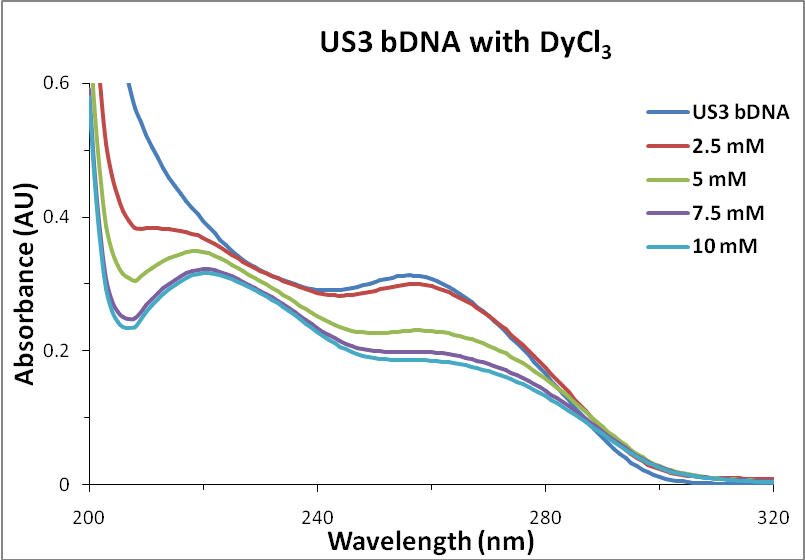

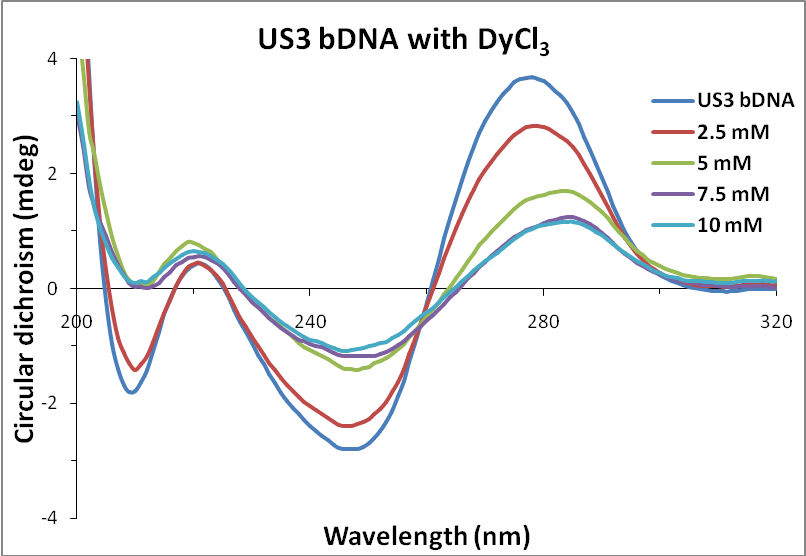


**Figure S5.** Absorbance and circular dichroism (CD) spectra of self-assembled bDNA US3 and its interaction with different concentrations of LaCl_3_, GdCl_3_, and DyCl_3_. US3 exhibit typical B-DNA conformation and DNA condensation was observed after increasing the concentration of REEs.

***
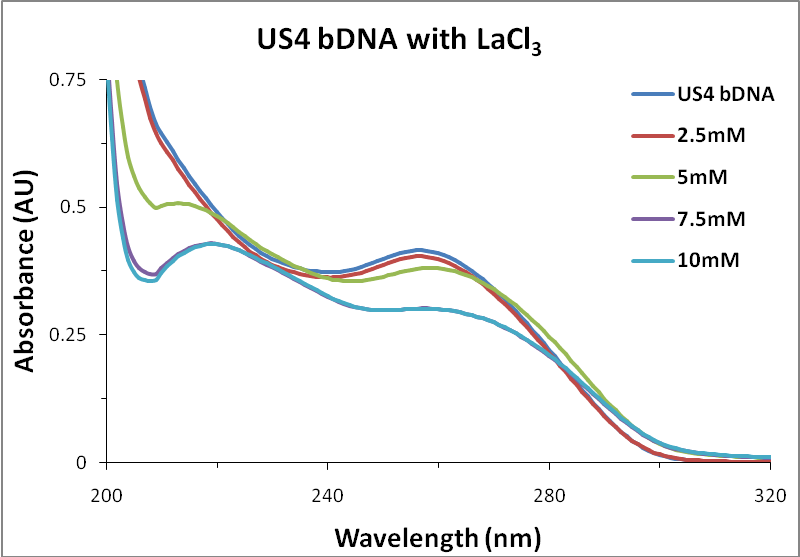

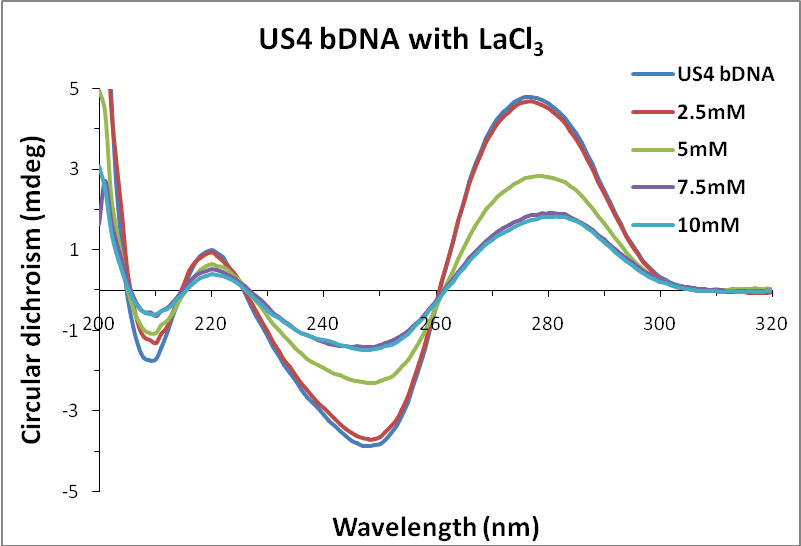

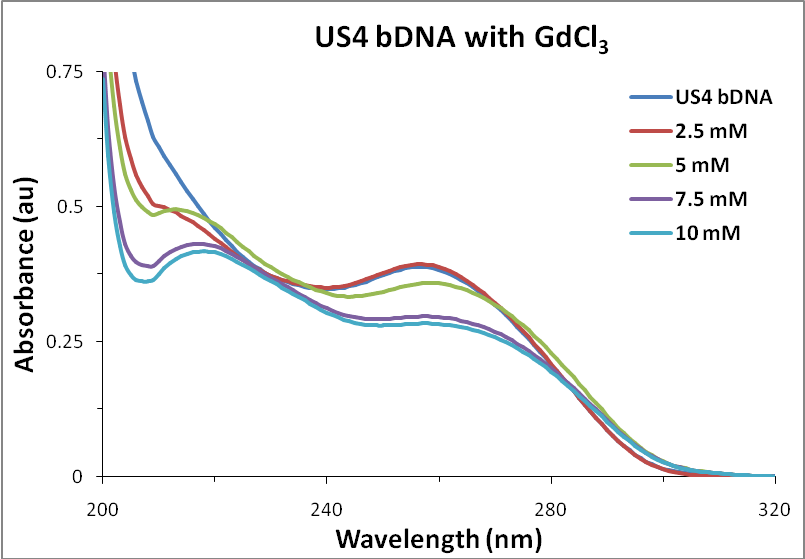

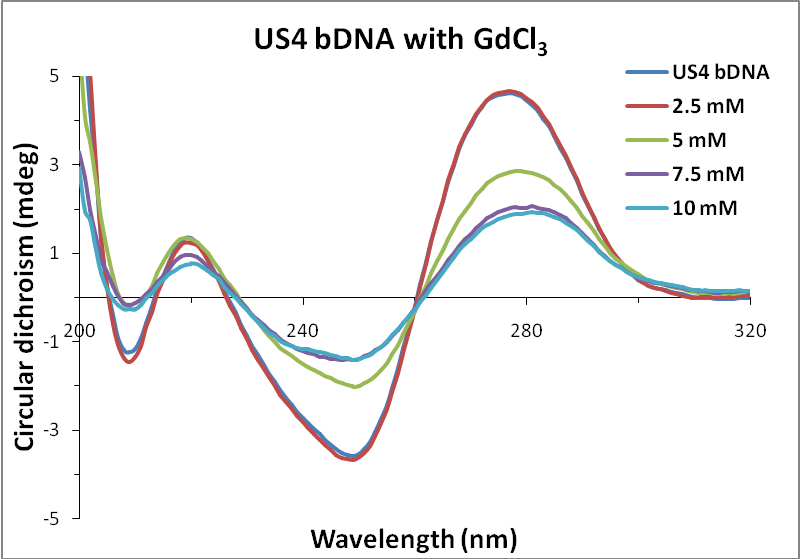

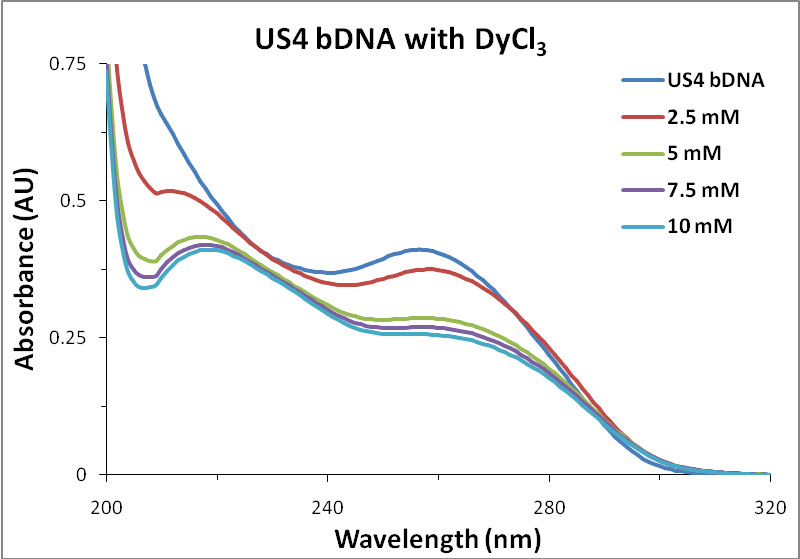

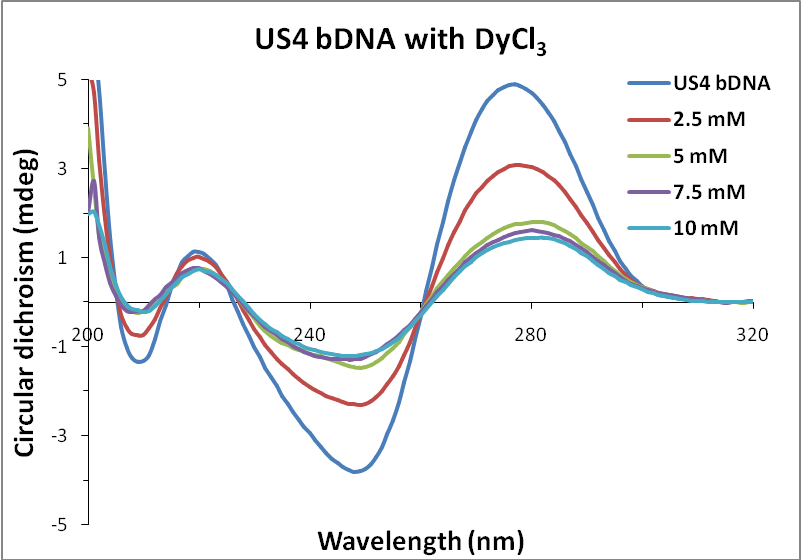
***

**Figure S6.** Absorbance and circular dichroism (CD) spectra of self-assembled bDNA US4 and its interaction with different concentrations of LaCl_3_, GdCl_3_, DyCl_3_. US4 exhibit typical B-DNA conformation and DNA condensation was observed after increasing the concentration of REEs.


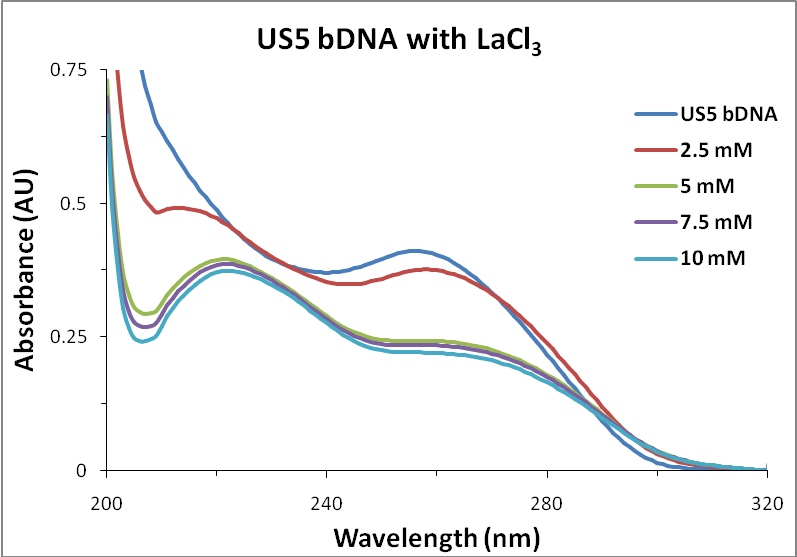

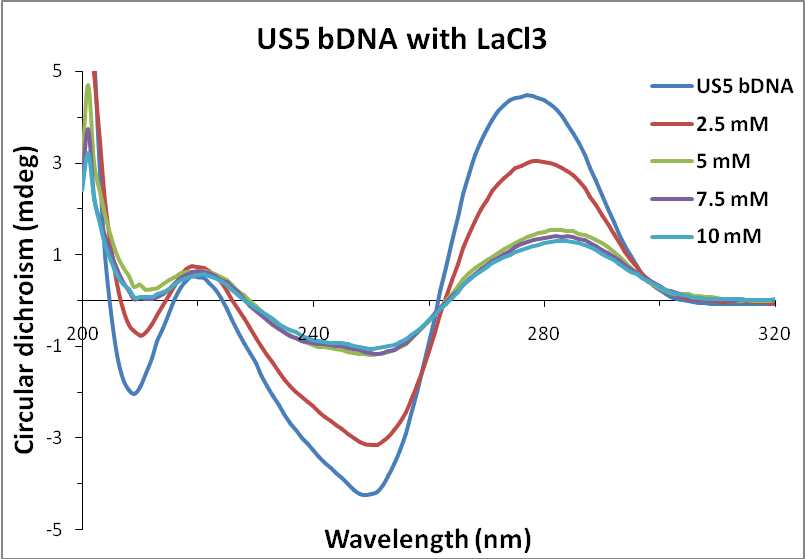

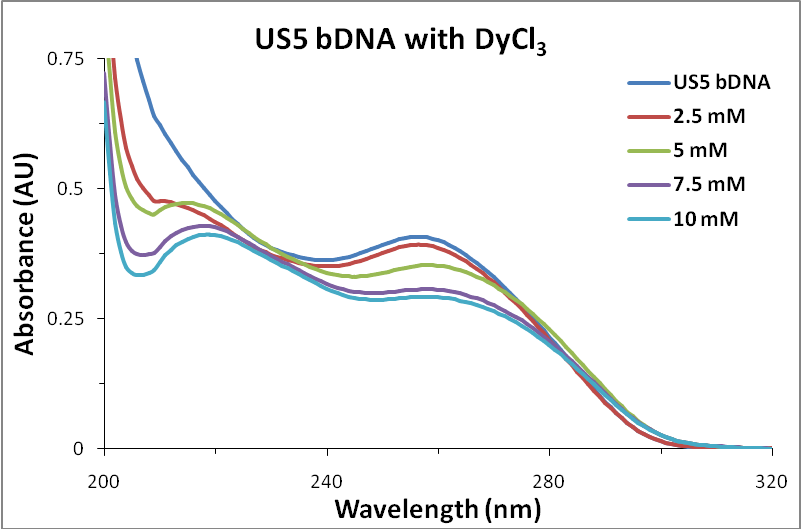

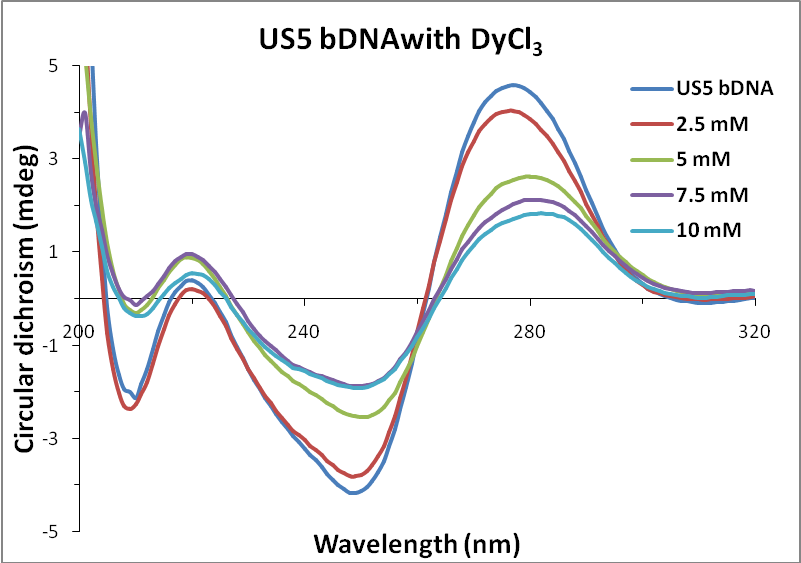

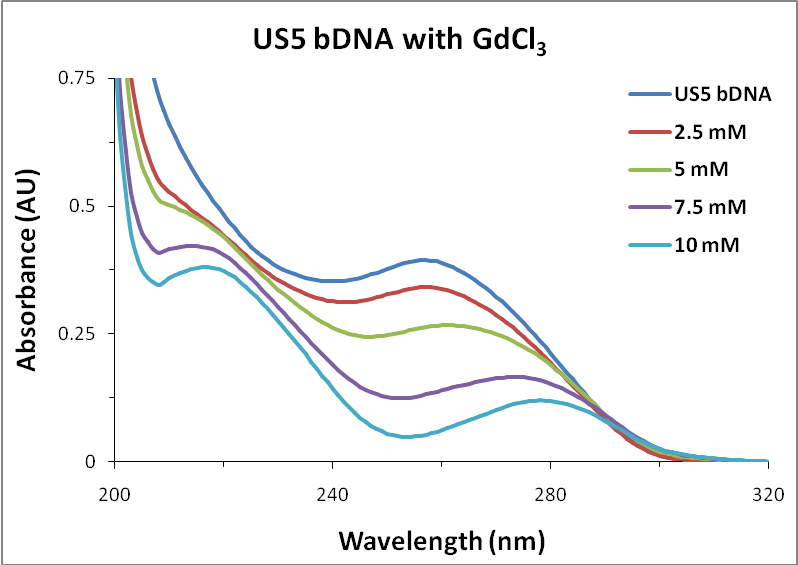

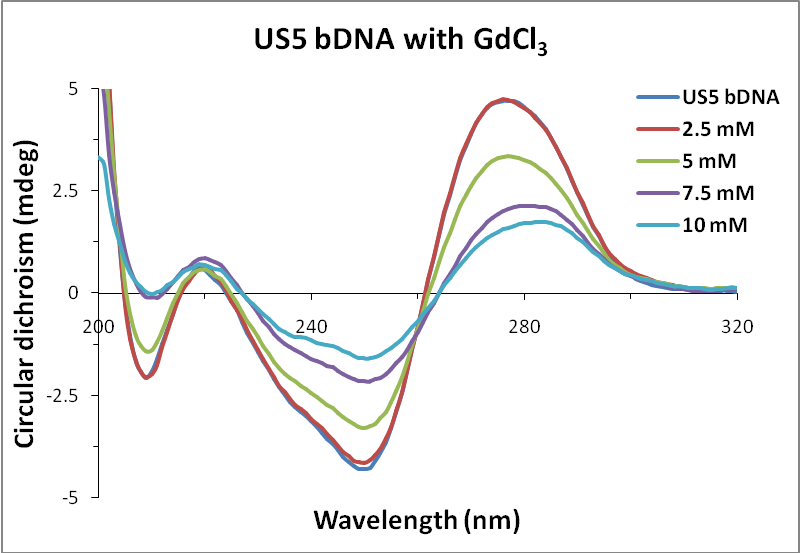


**Figure S7.** Absorbance and circular dichroism (CD) spectra of self-assembled bDNA US5 and its interaction with different concentrations of LaCl_3_, GdCl_3_, and DyCl_3_. US5 exhibit typical B-DNA conformation and DNA condensation was observed after increasing the concentration of REEs.


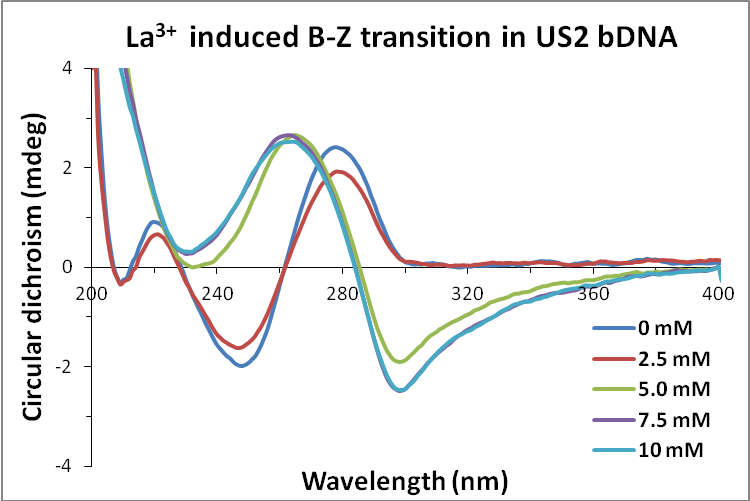

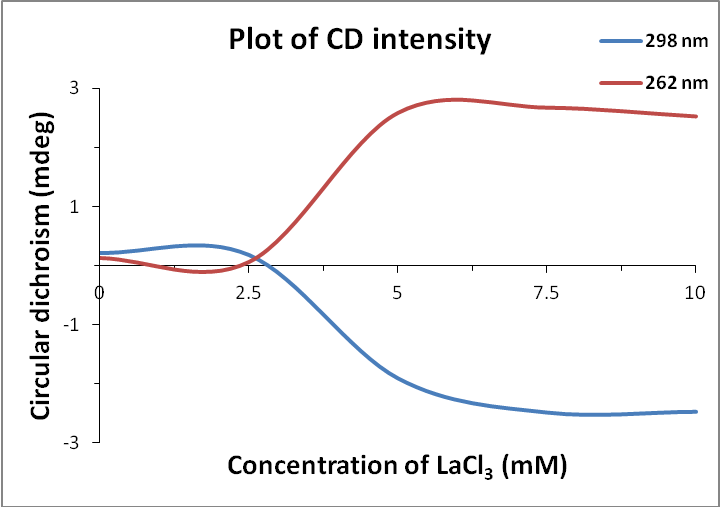


**Figure S8.** Typical B-Z transition of bDNA using LaCl_3_ (a), plot of CD intensity at 262 and 298 nm as a function of LaCl_3_ concentration (b). The data were adopted from the above graph.


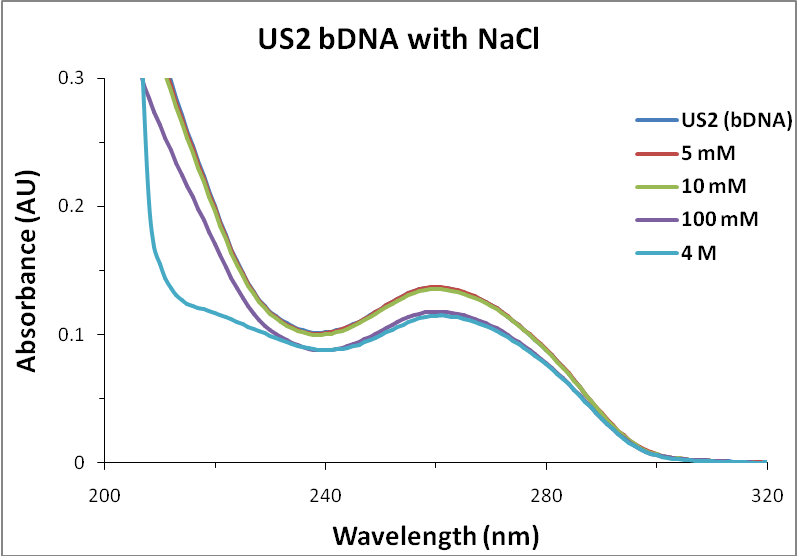

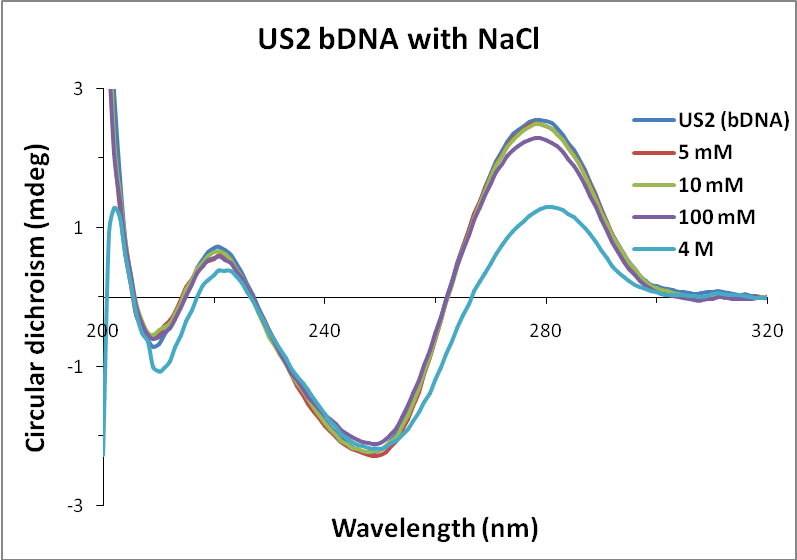

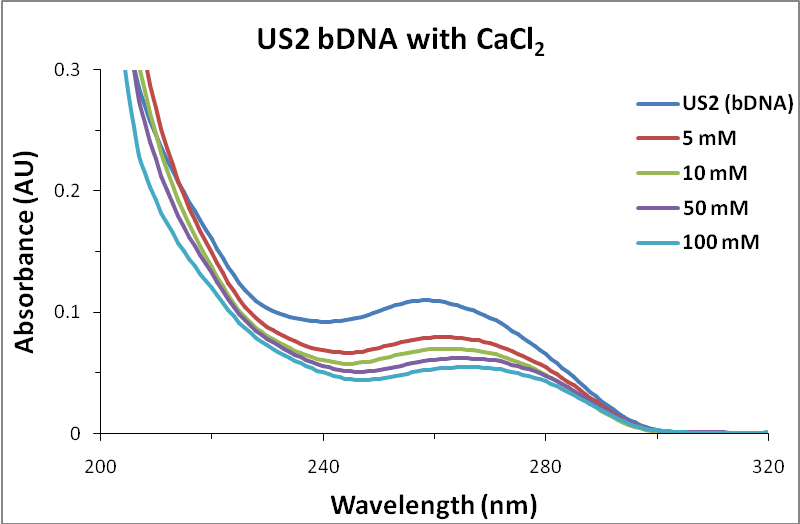

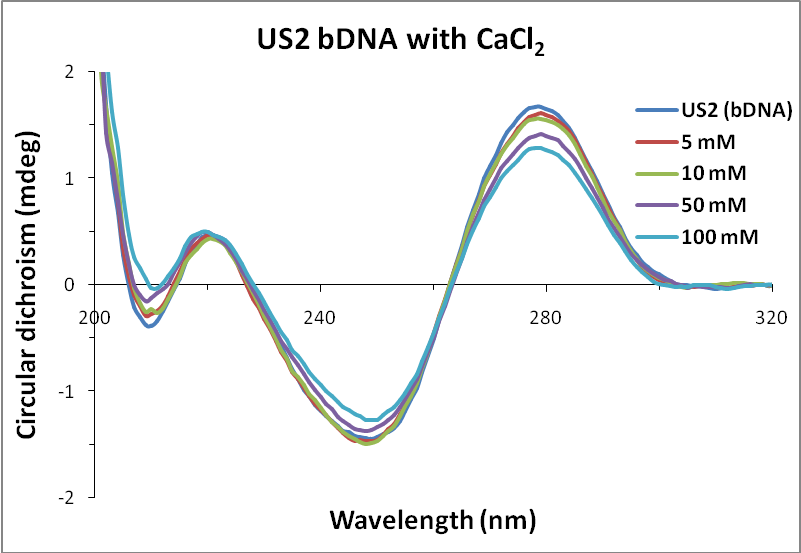

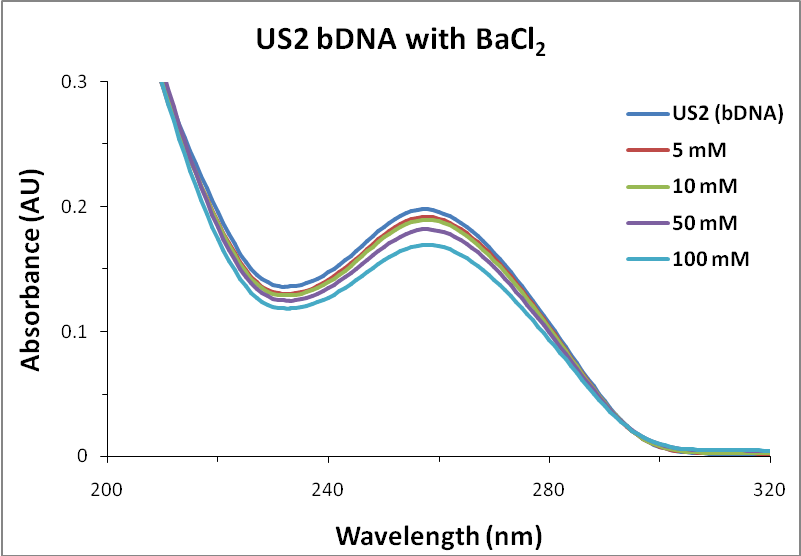

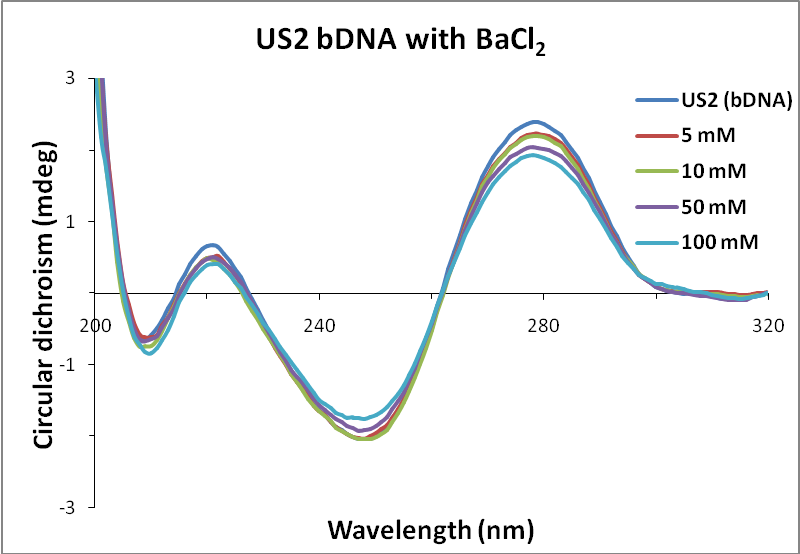


**Figure S9.** Interaction of US2 Y-shaped bDNA with monovalent (NaCl) and di-valent (CaCl_2_ and BaCl_2_). NaCl has no significant effect upto100 mM but showed a drastic decrease in intensity at ~280 nm with 4M NaCl. However, in case of di-valent cations (Ca^2+^ and Ba^2+^) slow condensation was observed up to 100 mM.


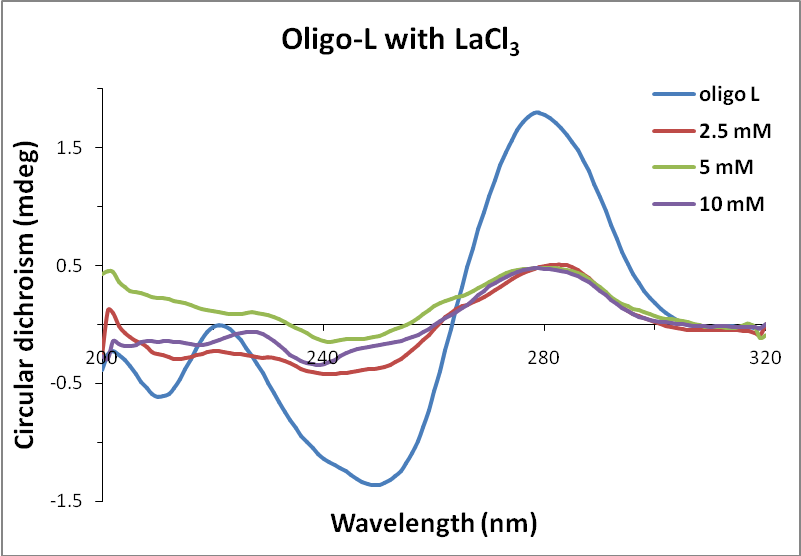

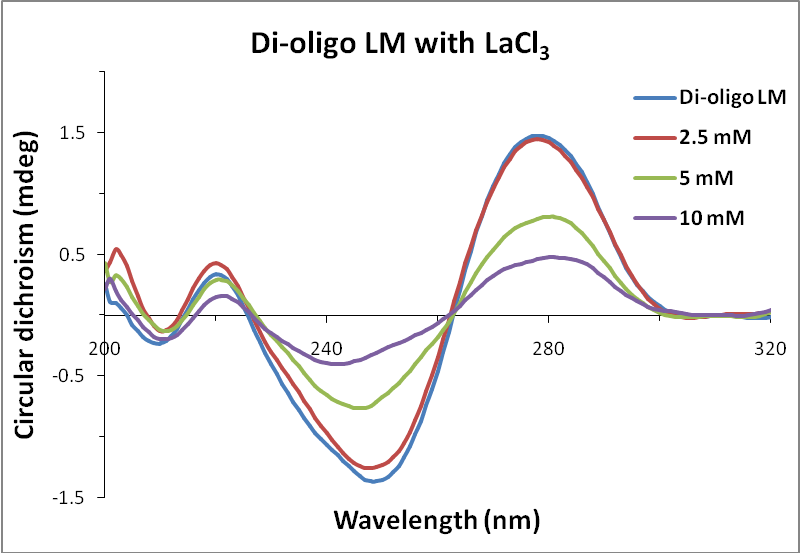

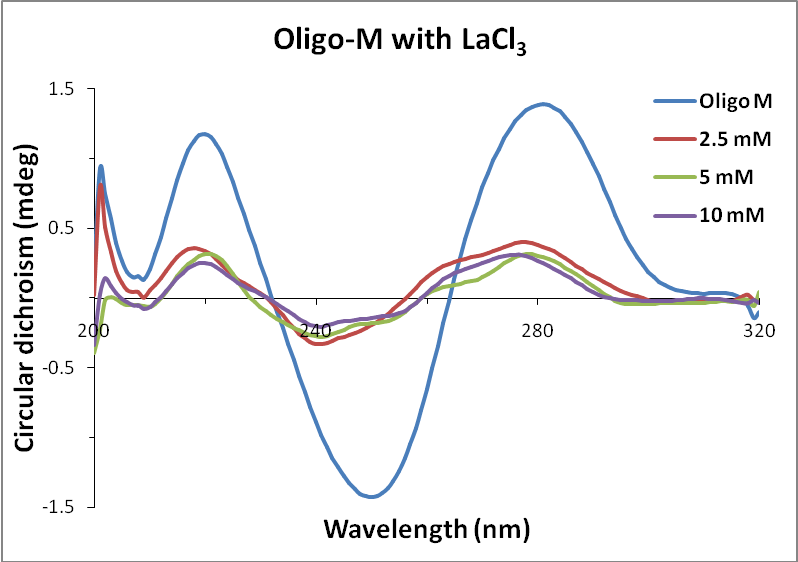

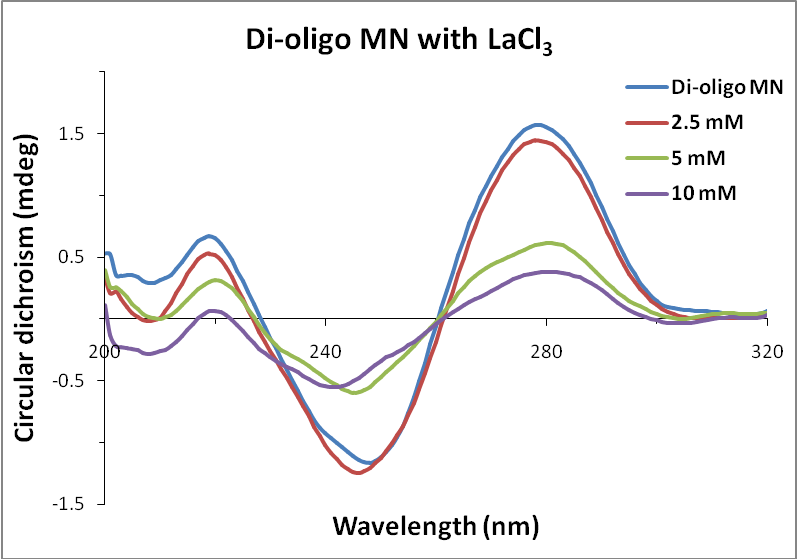

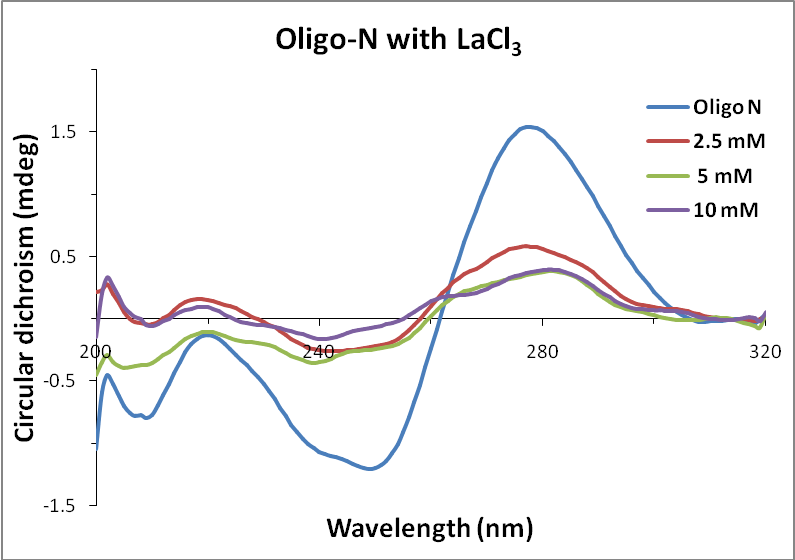

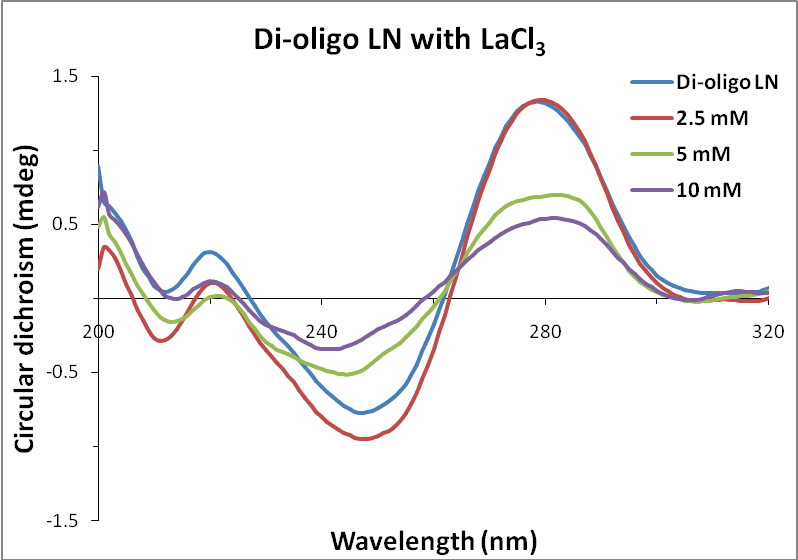


**Figure S10.** Circular dichroism (CD) spectra of individual (oligo) and dioligo complexes of US2 bDNA and their interaction with different concentrations of LaCl_3_. They all exhibit typical B-DNA conformation and DNA condensation was observed after increasing the concentration of LaCl_3_.


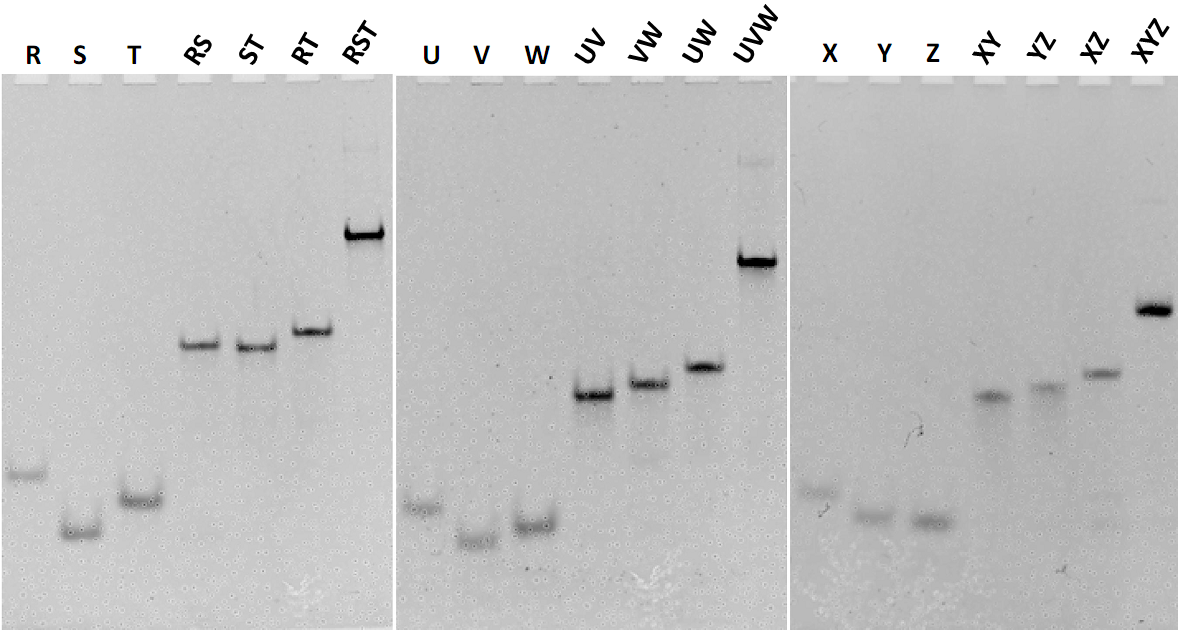


**Figure S11.** Characterization of the self-assembled bDNA structures using nPAGE (10%). The sample composition is labeled on the top of each lane. Gels showing the differential migration of individual oligos (R, S, T, U, V, W, X, Y, and Z), di-oligo complexes (RS, ST, RT, UV, VW, UW, XY, YZ, and XZ), and tri-oligo complexes (RST, UVW, and XYZ).


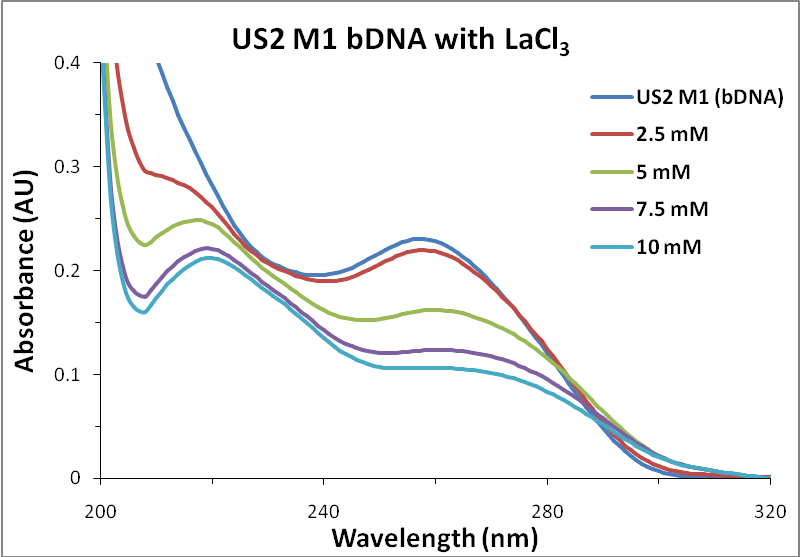

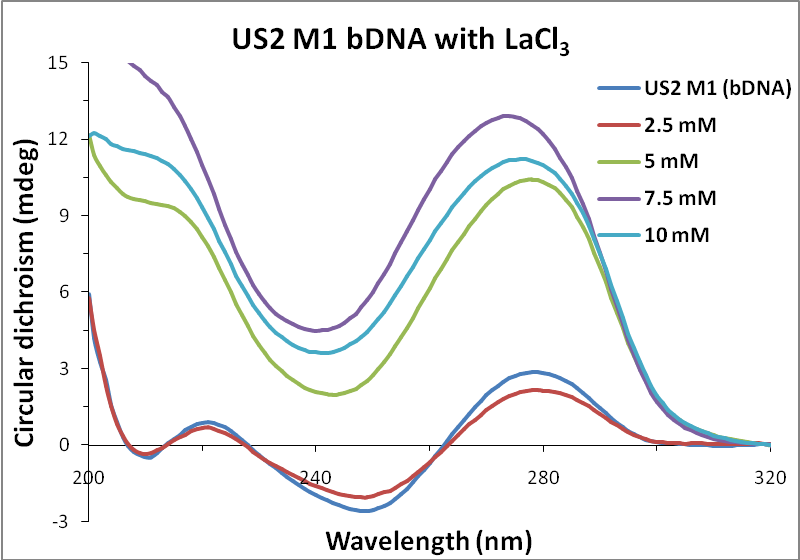

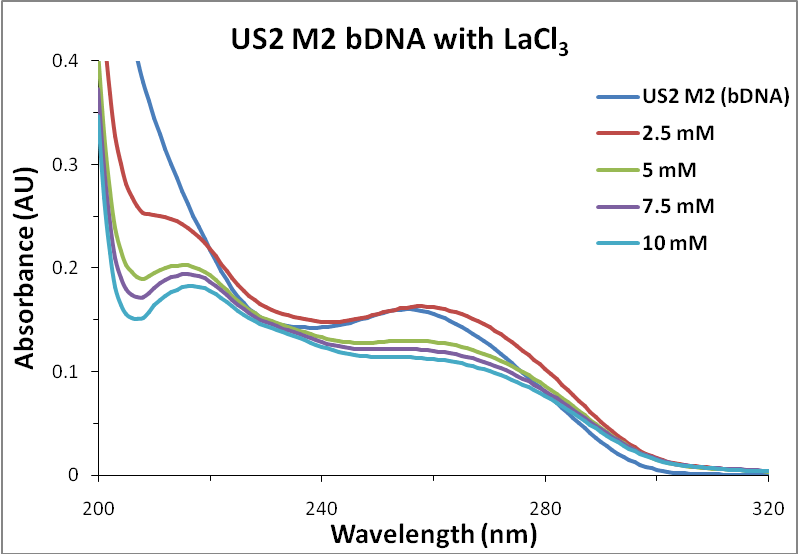

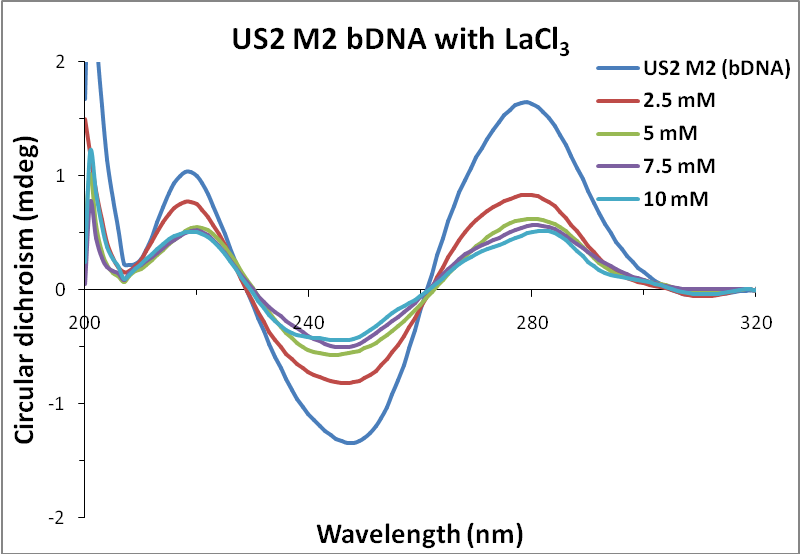

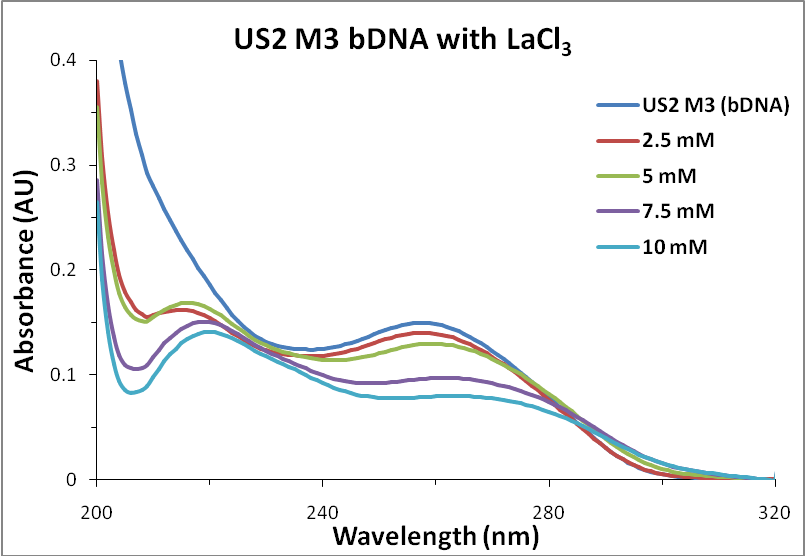

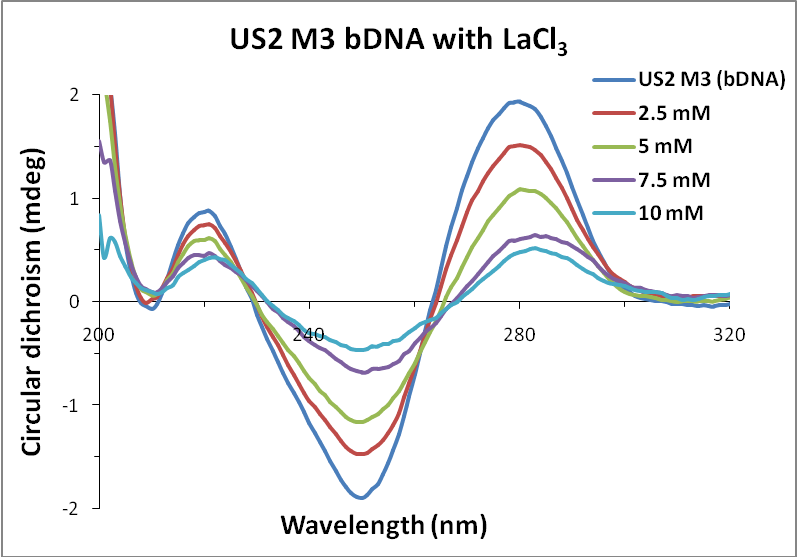


**Figure S12.** Interaction of modified Y-shaped structures derived from US2 with LaCl_3_. US2 M1 Y-shaped bDNA showed abnormal CD spectra when added with ≥ 5 mM LaCl_3_. However, US2 M2 showed a drastic condensation with addition of LaCl_3_. But US2 M3 structure showed uniform condensation pattern.


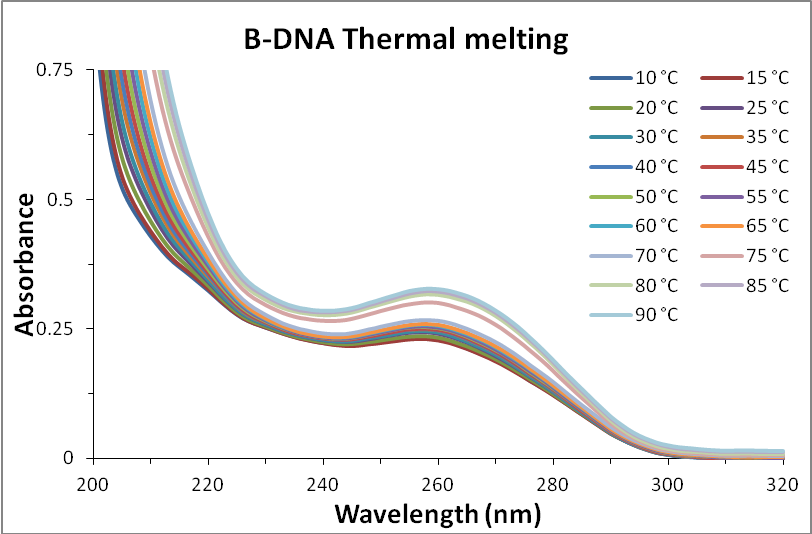

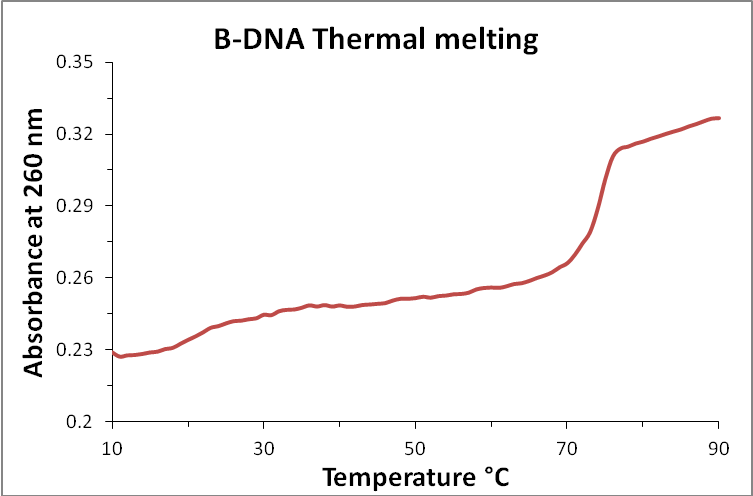

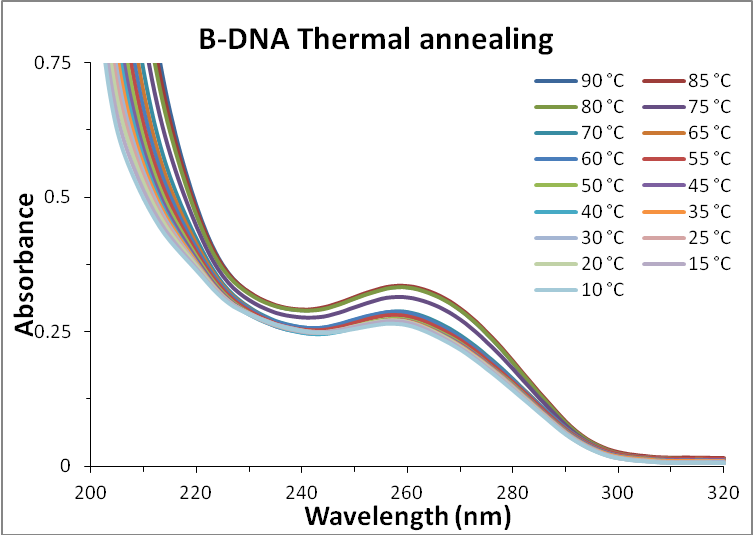

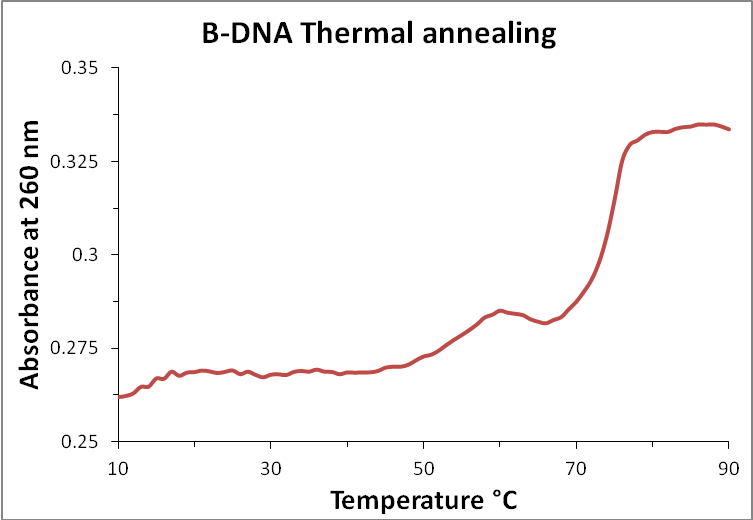


**Figure S13.** Melting and annealing curve of B-bDNA. Both during melting and annealing the T_m_ of B-bDNA was observed to be ~75°C.


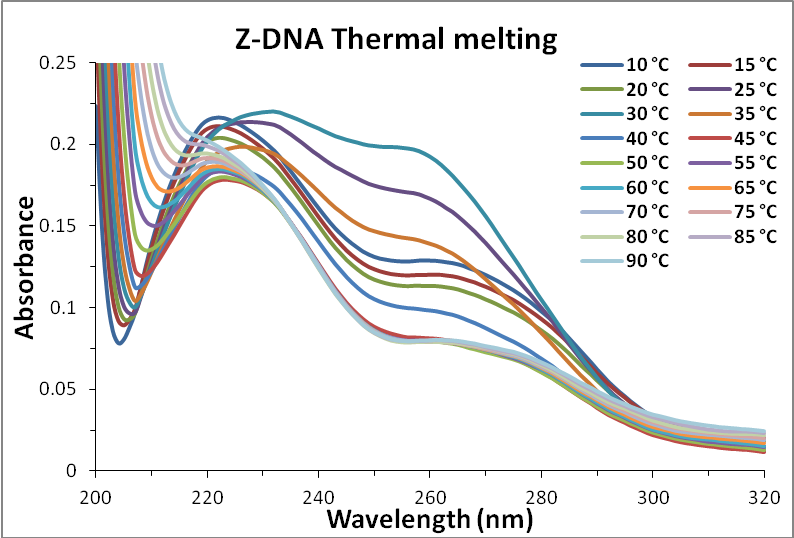

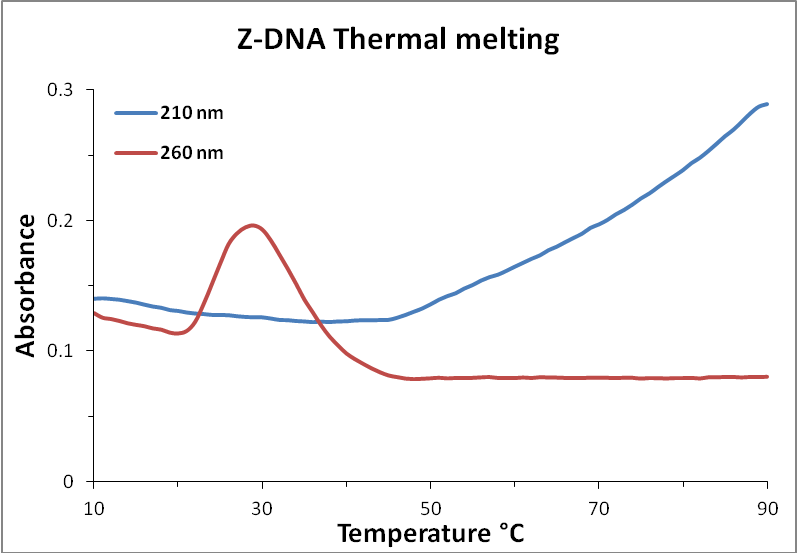

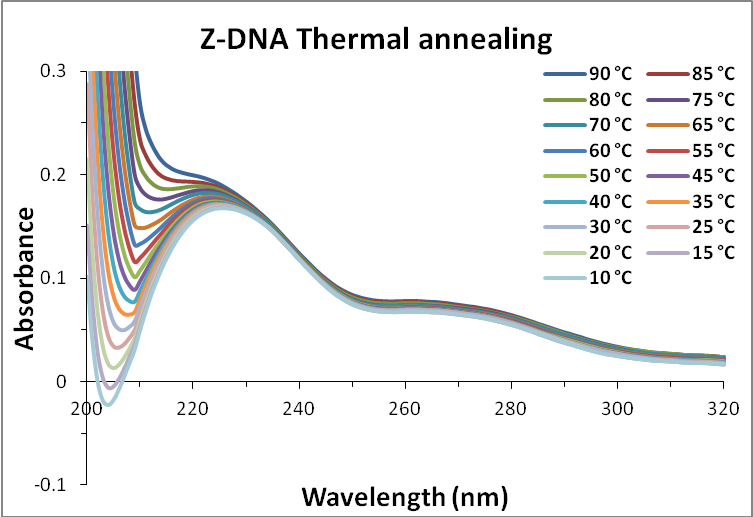

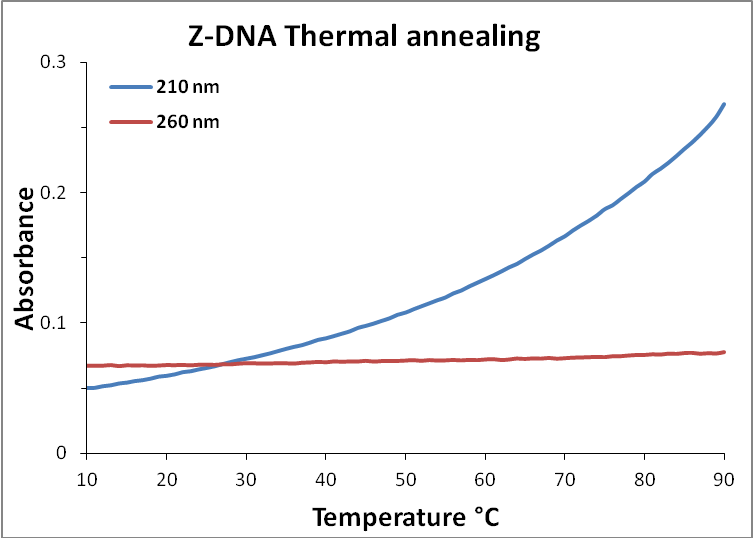


**Figure S14.** Melting and annealing curve of Z-DNA. The T_m_ of Z-DNA during melting was ~33°C at 260 nm. Nevertheless, during the process of denaturation the groove depth is decreasing with increase in temperature at 205 nm and was reversed during decreasing the temperature. No change in absorbance was observed at 260 nm in La^3+^-induced Z-DNA.
